# Assembly and annotation of the *Gossypium barbadense* L. ‘Pima-S6’ genome raise questions about the chromosome structure and gene content of *Gossypium barbadense* genomes

**DOI:** 10.1186/s12864-022-09102-6

**Published:** 2023-01-10

**Authors:** Ricardo A. Chávez Montes, Mauricio Ulloa, Tammy Biniashvili, Arik Zackay, Nir Kfir, Damar Lopez-Arredondo, Luis Herrera-Estrella

**Affiliations:** 1 Institute of Genomics for Crop Abiotic Stress Tolerance, Plant and Soil Science Department, Texas Tech University, 79409 Lubbock, TX USA; 2 Plant Stress and Germplasm Development Research, USDA-ARS, PA, CSRL, 79415 Lubbock, TX USA; 3 NRGene, Ness-Ziona, Israel; 4 Unidad de Genómica Avanzada/Langebio, Centro de Investigación y de Estudios Avanzados del Instituto Politécnico Nacional, 36821 Irapuato, Mexico

**Keywords:** Cotton genome, Pima cotton, Comparative genomics, Genome assembly

## Abstract

**Background:**

*Gossypium barbadense* L. Pima cotton is known for its resistance to Fusarium wilt and for producing fibers of superior quality highly prized in the textile market. We report a high-quality genome assembly and annotation of Pima-S6 cotton and its comparison at the chromosome and protein level to other ten *Gossypium* published genome assemblies.

**Results:**

Synteny and orthogroup analyses revealed important differences on chromosome structure and annotated proteins content between our Pima-S6 and other publicly available *G. barbadense* assemblies, and across *Gossypium* assemblies in general. Detailed synteny analyses revealed chromosomal rearrangements between Pima-S6 and other Pima genomes on several chromosomes, with three major inversions in chromosomes A09, A13 and D05, raising questions about the true chromosome structure of *Gossypium barbadense* genomes.

**Conclusion:**

Analyses of the re-assembled and re-annotated genome of the close relative *G. barbadense* Pima 3–79 using our Pima-S6 assembly suggest that contig placement of some recent *G. barbadense* assemblies might have been unduly influenced by the use of the *G. hirsutum* TM-1 genome as the anchoring reference. The Pima-S6 reference genome provides a valuable genomic resource and offers new insights on genomic structure, and can serve as *G. barbadense* genome reference for future assemblies and further support FOV4-related studies and breeding efforts.

**Supplementary Information:**

The online version contains supplementary material available at 10.1186/s12864-022-09102-6.

## Background

Cotton (*Gossypium* spp.) is the most important source of natural fiber that is grown in around 80 countries worldwide [1]. This genus comprises more than 50 extant species of which at least 45 are regarded as diploid, with 2n = 2x = 26, and at least seven as allotetraploid, with 2n = 4x = 52 [2–7]. The common allotetraploid AD genome architecture of all extant 52 chromosomes in *Gossypium* species is thought to have originated from a relatively recent, ~ 1.0 million years ago, polyploidy event of monophyletic origin [5]. Within the allopolyploid species, *G. barbadense* is one of the species with the most recent divergence (~ 0.20 Ma) with *G. darwinii* L. (AD_5_). *G. barbadense* is indigenous to the northern part of South America and extends into Mesoamerica and the Caribbean, and is also known as Pima, Long Staple, Sea Island, Egyptian, or Tanguis cotton [5, 7, 8].

In the U.S., Upland and Pima cotton account for around 95.5% and 4.5%, respectively, of total fiber production. However, Pima cotton is highly valued in the premium textile market because of its superior fiber fineness, length, and strength qualities. Pima differs from Upland cotton in numerous traits such as yield, adaptation and growth habits, among others. Specifically, Pima S-6 was released in 1984 [9], and was used as the source of resistance of highly pathogenic *Fusarium oxysporum* f. sp. *vasinfectum* (FOV) race 4 in the first commercial Pima varieties and public germplasm releases in the U.S. [10–14] (see Materials and Methods). FOV is a soil borne fungal pathogen that threatens cotton production around the world, and for over a decade, FOV4 strain has adversely impacted cotton production in the U.S., causing plant wilt and death [10–12]. Since Pima S-6 identification as a source of FOV4-resistance, it was subjected to several cycles of evaluations and selections under FOV4 infested fields to increase its uniformity and the level of resistance [10–14]. This new selection source was renamed Pima-S6. Therefore, genomic resources that help further improve Pima-S6 through modern techniques are highly valuable.

Considerable progress has been made toward the development of new cotton genomic resources. Published cotton genomes of diploid ancestors (A_1_, A_2_, D_5_), wild polyploid [*G. tomentosum* (AD_3_), *G. mustelinum* (AD_4_), and AD_5_ *G. darwinii*] and tetraploid cotton (AD_1_ Upland, AD_2_ Pima) are providing the opportunity to better understand the history of cotton domestication and genome structure [5, 15–22]. Cotton genomic resources are also crucial to facilitate the identification of genes and alleles important for crop improvement, the identification of recombination events and selection signatures important not only for yield and fiber quality, but also for resistance to root-knot-nematode (*Meloidogyne incognita*) [23] and FOV4 [12]. However, much of the emphases of high-quality genome assemblies and structural genetic and gene expression variants have been concentrated in *G. hirsutum* and genomic information of the cultivated G. *barbadense* Pima is still limited.

Here we report a chromosome level assembly and annotation of *G. barbadense* Pima-S6. We conducted multiple comparisons to previously published *Gossypium* genomes and found important variations in genome structure and annotated proteins. The Pima-S6 reference genome provides valuable genomic resources for dissecting FOV4 resistance genes and for the improvement of the cotton crop.

## Results

### Sequencing, assembly, and annotation of *G. barbadense* Pima-S6 genome

Using the DeNovoMAGIC^™^ platform, short sequencing reads from shotgun, mate-pair and Gemcode libraries (see Materials and Methods) were assembled into 19,148 scaffolds, and *G. barbadense* Pima-S6 pseudo-chromosomes were then reconstructed based on their alignment and anchoring to the *G. hirsutum* TM-1 reference genome version UTX_v2.1 (available at Phytozome) [5]. Assembly results are summarized in Table 1. A 2,301,422,177 bp assembly was obtained, of which 97.52% (2,244,350,239 bp) was assigned to 26 pseudo-chromosomes, 0.001% (23,063 bp) mapped to unplaced TM-1 contigs, 2.54% (57,048,875 bp) unmapped to TM-1, and 1.46% gaps (Table 1). The final 2.3 Gb Pim-S6 assembly was annotated using MAKER-P [24, 25] (see Materials and Methods). MAKER-P predicted 88,343 genes, 75,419 of which have a predicted CDS sequence that translated into proteins with evidence of homology to known proteins (Table 1). The distribution of Annotation Edit Distance scores, a measure of the goodness of fit of an annotation to the evidence supporting it [25–27], is presented in Supplementary Fig. 1.

**Table 1:** Pima-S6 genome assembly and annotation statistics

Statistics	Value
Number of scaffolds	19,148
Scaffold N50 (bp)	34,473,498
Longest scaffold (bp)	75,397,024
Scaffold assembly size (bp)	2,301,208,477
26 chromosomes size (bp)	2,244,350,239
Genome in chromosomes (and gaps, %)	97.5 (1.46)
GC content (%)	34
Number of genes predicted by MAKER-P	88,343
Number of predicted genes with evidence of protein homology (in A genome; in D genome)	75,419 (36,158; 37,431)
Number of genes with GO annotation (%)	64,086 (85%)
Number of genes without evidence of protein homology, but with evidence of expression in RNA-seq data	1,965

The number of 75,419 genes, which we used as the final annotation for Pima-S6, is in accordance with the ~ 75,000 genes reported for the *G. barbadense* ‘Pima 3–79’ HGS (hereafter Pima 3–79 HGS, available at Phytozome, with 74,561 genes) [5], ‘Hai7124’ (hereafter Hai7124, available at cottongen, with 75,071 genes) [15] or TM-1 (with 75,376 genes) genome assemblies. Pima-S6 RNA-seq data from two sequencing libraries, one from leaves and one from roots (Supplementary Table 1) showed that of the 75,419 genes with protein homology, 37,823 genes had evidence of expression (expression value > 1 TPM) in leaves and 51,071 in roots (Supplementary Fig. 2). From the total genes expressed in leaves, 17,198 came from the A subgenome, whereas 17,634 came from the D subgenome. In the case of genes expressed in roots, 23,101 genes came from the A subgenome and 23,803 genes came from the D subgenome. Of the 12,924 genes that were filtered due to lack of protein homology evidence, 1,965 had evidence of expression in both leaf and root samples.

**Fig. 1:**
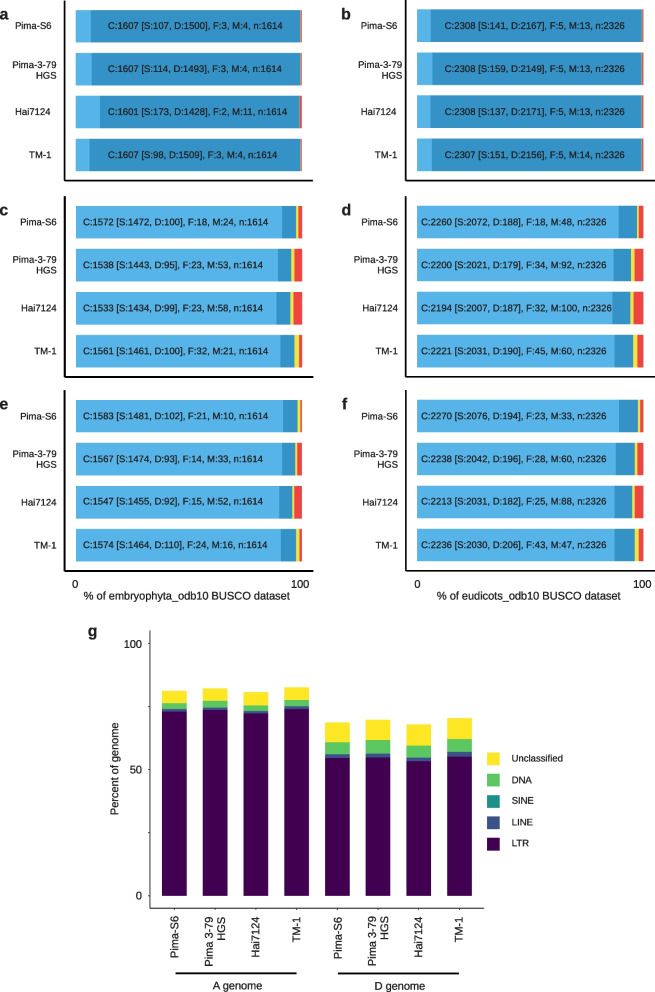
*G. barbadense* Pima-S6, Pima 3–79 HGS and Hai7124, and *G. hirsutum* TM-1 BUSCO statistics and repeats content. Barplots show the genome (**a**,** b**), A subgenome proteins (**c**,** d**) and D subgenome proteins (**e**, **f**) BUSCO statistics with the embryophyta_odb10 and eudicots_odb10 dataset statistics [light blue: complete (C) and single (S); blue: complete and duplicated (D); yellow: fragmented (F); red: missing (M)]. Datasets contain 1614 (embryophyta_odb10) and 2326 eudicots_odb10) entries (n). Genome BUSCO statistics indicate that the Pima-S6 genome assembly is complete and comparable to those of Pima 3–79 HGS, Hai7124 and TM-1. Protein BUSCO statistics show an improvement when compared to Pima 3–79 HGS, Hai7124 or TM-1. (**g**) Transposons content in these four genome assemblies. LTR: Long Terminal Repeat; SINE: Short Interspaced Elements; LINE: Long Interspaced Nuclear Element

We then proceeded to compare and analyze the completeness of Pima-S6, Pima 3–79 HGS, Hai7124, and TM-1 assemblies and annotation elements using BUSCO [28] and the content of repetitive elements (Fig. 1). All four genome assemblies had over 99% completeness evaluated with the embryophyta_odb10 and eudicots_odb10 datasets, with most genes marked as duplicated, as expected, because of the tetraploid nature of these cotton genomes (Fig. 1a and b). A BUSCO analysis of the annotated proteins showed that all four genome annotations are complete. Pima-S6 has a slightly better score (97.4% and 98.1% for embryophyta_odb10, 97.2% and 97.6% for eudicots_odb10 of complete BUSCOs for the A and D subgenomes, respectively), than Pima 3–79 HGS (95.3% and 97.1% embryophyta_odb10; 94.6% and 96.2% eudicots_odb10), Hai7124 (94.9% and 95.8% embryophyta_odb10; 94.3% and 95.1% eudicots_odb10) or TM-1 (96.7% and 97.5% embryophyta_odb10; 95.5% and 96.2% eudicots_odb10) (Fig. 1c-f).

The composition of the different classes of repetitive elements in our Pima-S6 assembly is also comparable to that of other cotton assemblies, with over 70% of the A subgenome and over 50% of the D subgenome annotated as repeat sequences (Fig. 1 g). All four assemblies have a similar distribution of Long Terminal Repeat (LTR) retrotransposon families. Interestingly, content of LTR classes Ty1/copia/Ivana, Ty1/copia/Tork, Ty3/gypsy/chromovirus/chromo-outgroup of the A subgenome in Pima S-6 is higher as compared to other Pima, but similar to TM-1; Ty3/gypsy/non-chromovirus/OTA/Tat/Ogre content was also higher in Pima-S6 compared to other Pima, but similar to TM-1; Ty3/gypsy/non-chromovirus/OTA/Tat/Ogre content was also higher in Pima-S6 as compared to other Pima; whereas Ty3/gypsy/chromovirus/CRM was higher in Pima-S6 than any other cotton entry (Supplementary Fig. 3a). In reference to the D subgenome, content of three LTR retrotransposon families, Ty1/copia/Tork, Ty3/gypsy/chromovirus/CRM, and Ty3/gypsy/chromovirus/Tekay were higher in Pima-S6 than any other cotton entry (Supplementary Fig. 3b; Supplementary Table 2). In addition, in both subgenomes, the Ty3/gypsy/non-chromovirus/OTA/Tat/Ogre content was higher in TM-1 than any of the Pima genomes (Supplementary Fig. 3a, b).

#### Chromosome-level structural variations between Pima-S6 and TM-1

After comparing our Pima-S6 genome to other *Gossypium* genomes at the macro level, we proceeded to search for structural genome variations between them. We first identified chromosomal structural variations, i.e., synteny, inversions, translocations, and duplications, between Pima-S6 and TM-1. Major (length > 1 Mbp) inversions, duplications or translocations are present in chromosomes A01, A02, A03, A04, A06, A07, A11, A12, A13, D03, D05, D06, D07, D08 and D12 (Supplementary Fig. 4). We then performed a detailed analysis to determine whether the end points of the structural variations fall inside an assembled contig, suggesting that they were real, or at the end of contigs, suggesting that they could be contig placement artifacts. We analyzed in more detail the boundaries of the Pima-S6 vs. TM-1 major inversions in four chromosomes, A11, A03, A12 and D12 (Supplementary Fig. 5). In all cases, we identified scaffolds that contain both syntenic and inverted regions and traverse the syntenic to inversion boundaries. Chromosome A11 contains three major inversions (Supplementary Fig. 5a). Inversion 1 (Inv1) is a ~ 11.8 Mbp inversion whose boundaries traverse unambiguously assembled regions, strongly suggesting that the inversion is real and not due to an assembly artifact (Supplementary Fig. 5a). Inversion 2 (Inv2) starts at an unambiguously assembled region and ends at the beginning of a 1,000 bp N stretch followed by 535 bases of unambiguously assembled sequence, which themselves are an inversion-duplication nested within Inv2 on the TM-1 side (Supplementary Fig. 5b). The 1,000 bp N stretch most likely represents a short read-unresolved assembly region, while placement of the 535 bp during assembly was supported by linkage evidence. Re-alignment of the mate pair genomic sequencing reads showed that uniquely aligned reads, i.e., alignments with the filter bowtie2 XS flag null and not duplicated, have one mate aligning to the 535 bp region and the second mate several thousand bp upstream, confirming the linkage evidence (Supplementary Fig. 5c). Inversion 3 (Inv3) starts at the end of a 100 bp gap between scaffolds and ends at an unambiguously assembled region (Supplementary Fig. 5a, b). These analyses strongly suggest that Inv2 and Inv3 are also real and not assembly artifacts. This conclusion is further supported by the fact that the Inv2 to Inv3 region is fully contained within a syntenic region between Pima-S6 and *G. barbadense* Pima 3–79 HAU.2 (see next section). The boundaries of the major inversions in chromosomes A03, A12 and D12 were also analyzed (Supplementary Fig. 5c, d). They traverse unambiguously assembled regions, except for a boundary of the first inversion of chromosome A03, which is followed by a 10 bp N stretch and a 1,532 bp inversion-duplication of unambiguously assembled region that aligns to a region on chromosome A11 on the TM-1 side.

All these observations strongly suggest that the major inversions between Pima-S6 and TM-1 in chromosomes A03, A11, A12 and D12 are real and not due to assembly artifacts. Pima-S6 chimeric scaffolds were split during pseudo-chromosome reconstruction, but further splitting, reorienting, and moving of Pima-S6 scaffolds to accommodate TM-1 chromosomal structure would imply disregarding the sequencing read evidence subtending them. These Pima-S6 scaffold to TM-1 chromosome synteny analyses do support the validity of the identified chromosomal rearrangements between the Pima-S6 and TM-1 assemblies.

#### Chromosome structural variations between Pima-S6 and other published *G. barbadense* assemblies

After validating major structural differences between Pima-S6 and *G. hirsutum* TM-1, we compared the chromosome structure of the Pima-S6 assembly against other publicly available *G. barbadense* assemblies: Pima 3–79 HAU.1 (hereafter Pima 3–79 HAU.1, available at cottongen) [22], Pima-3-79 HAU.2 (hereafter Pima 3–79 HAU.2, available at cottongen) [20], Pima-3-79 HGS, Pima90 [29], and Hai7124. We first obtained the chromosome lengths for each assembly. Pima-S6 chromosomes are on average 4% (3.4 Mb) longer than the corresponding chromosomes from Pima 3–79 HAU.2, Pima 3–79 HGS, Pima90 or Hai7124 assemblies (Supplementary Table 3). The slightly longer Pima-S6 chromosomes could be due to a better assembly, a lower number of gaps, or true small differences between Pima cotton genotypes caused by differences in repetitive elements content.

**Fig. 2:**
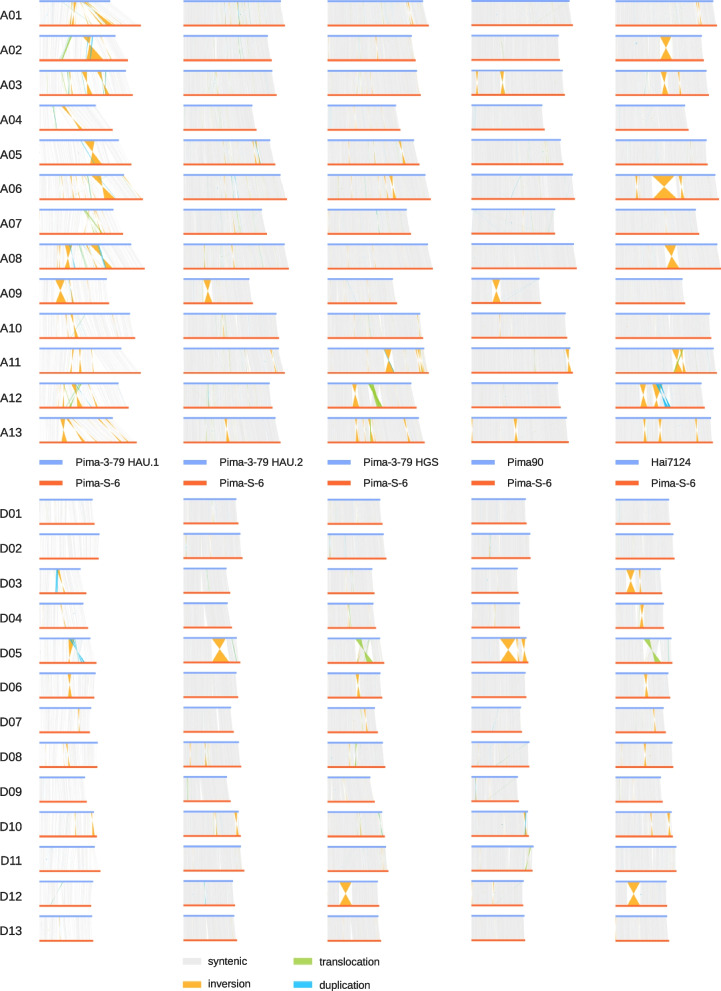
Synteny plots between Pima-S6 and Pima-3-79 HAU.1, Pima3-79 HAU.2, Pima-3-79 HGS, Pima90 and Hai7124. In these synteny plots, the reference genome is represented by blue horizontal lines and the query genome by red horizontal lines. Vertical lines represent syntenic (gray), inverted (orange), translocated (green) and duplicated (blue) regions. Chromosome ids are indicated on the left side of the plots. The 26 Pima-S6 chromosomes were aligned using minimap2 versus the 26 chromosomes of the Pima-3-79 HAU.1, Pima-3-79 HAU.2, Pima-3-79 HGS, Pima90 or Hai7124 genome assemblies, and synteny analysis was carried out using syri

We then identified chromosomal structural variations, i.e., synteny, inversions, translocations, and duplications, between our Pima-S6 assembly and the Pima 3–79 HAU.1, Pima 3–79 HAU.2, Pima 3–79 HGS, Pima90 and Hai7124 assemblies (Fig. 2). We observed that no synteny plot between Pima-S6 and any other *G. barbadense* assembly is identical, and important chromosomal structural variations exist between most chromosomes and across chromosomal comparisons, except for chromosomes D01, D02, D11, and D13. First, a comparison of the HAU.1 and HAU.2 plots vs. Pima-S6 shows a reduction in chromosome structural variation and suggests an improvement in contig placement during Pima 3–79 HAU.2 assembly. In the HAU.1 comparison there are 42 inversions, 3 inversion-duplications, 1 inversion-translocation and 1 translocation with length of 1 Mbp or longer, while in the HAU.2 comparison there are only 12 inversions with length of 1 Mbp or longer (Fig. 2). We then compared the Pima-S6 vs. Pima 3–79 HAU.2 and Pima HGS plots, and the differences between the two were intriguing, since both assemblies were obtained for the same cultivar, Pima-3-79. The Pima-S6 vs. Pima 3–79 HGS plot has 21 structural variations with length of 1 Mbp or longer: 15 inversions, 2 inversion-translocations and 4 translocations. Most of the inversions, duplications, and translocations in the Pima 3–79 HGS plot are not visible in the Pima-3-79 HAU.2 plot, e.g., those present in chromosomes A06, A11, A12, D06 or D12. It is important to mention that the Pima-3-79 HAU.2 and Pima-3-79 HGS assemblies share the same cultivar-pedigree seed source, Pima 3–79. The next plot, PimaS6 vs. Pima90, has 10 inversions and 1 inversion-translocation with length of 1 Mbp or longer, and is similar to the Pima-S6 vs. Pima-3-79 HAU.2 plot, with the two larger inversions in chromosomes A09 and D05 common to both plots and absent from the Pima-S6 vs. Pima-3-79 HGS plot, and a smaller inversion in chromosome A13 common to the Pima-3-79 HAU.2, Pima-3-79 HGS and Pima90 plots. We then examined the Pima-S6 vs. Hai7124 plot. In this comparison, there are 30 structural variations, 24 inversions, 4 inversion-duplications, 1 inversion-translocation and 1 translocation with length of 1 Mbp or longer. Some of these structural variations are also visible in the Pima 3–79 HGS plot, e.g., the inversions in chromosome A11, A12, A13 and D12, and the translocation in chromosome D05, while others only are present in the Pima 3–79 HGS comparison, such as the inversions in chromosomes A06 and A08 (Fig. 2).

It seems unlikely that Pima-3-79 HGS would have a chromosome structure more similar to Hai7124, a different cultivar, than Pima-3-79 HAU.2, the same cultivar. The similarities between the HAU.2 and Pima90 plots, the presence of chromosomal structural variations in the Pima 3–79 HGS plot that are absent on the HAU.2 and Pima90 plots, and the presence of common structural variations between the Pima 3–79 HGS and Hai7124 plots, would suggest that the placement of some contigs of the *G. barbadense* Pima-3-79 HGS assembly might be incorrect. These intriguing findings, and the availability of the Pima 3–79 unplaced contigs under BioProject accession PRJNA516411, persuaded us to further investigate these phenomena by re-scaffolding the *G. barbadense* Pima 3–79 HGS assembly.

#### Re-scaffolding of the Pima 3–79 HGS genome and synteny plots re-analysis

Pseudo-chromosome reconstruction of the Pima-3-79 HGS assembly used a combination of Hi-C data and synteny versus a previous TM-1 assembly [30]. If the chromosome structural variations we observed between the Pima-S6 and Pima 3–79 HGS assemblies are due to the excessive influence of *G. hirsutum* as the scaffold placement reference, a re-scaffolding of the unplaced Pima 3–79 HGS contigs using Pima-S6 as a reference should eliminate most chromosomal rearrangements. To test this, we used Pima-S6 genome as the reference to re-scaffold the 4,748 unplaced Pima 3–79 HGS contigs using the RagTag pipeline [31], and the resulting re-assembly was named Pima 3–79 RagTag. This re-scaffolding recovers the 26 pseudo-chromosomes, with a total size of 2,170,092,153 bp versus 2,130,186,492 bp for Pima 3–79 HGS (Supplementary Table 3). Of the 4,748 contigs, only 774 (totaling 24,244,590 bp) remained unplaced in Pima 3–79 RagTag, versus 2,022 unplaced contigs (totaling 65,618,451 bp) in the Pima 3–79 HGS assembly. Re-scaffolding of the Pima 3–79 HGS unplaced contigs resulted in a slightly better assembly of the 26 chromosomes of Pima 3–79 RagTag with an average of 1.8% longer chromosomes than their corresponding original Pima 3–79 HGS chromosomes (Supplementary Table 3).

A synteny analysis between the Pima-S6 and Pima 3–79 RagTag assemblies (Supplementary Fig. 4c) now shows that both genomes have very similar chromosome structures, except for a ~ 4 Mbp inversion in chromosome A01. This most likely indicates that Pima 3–79 HGS 26 pseudo-chromosome reconstruction was excessively influenced by the use of *G. hirsutum* as the anchoring reference. The influence of *G. hirsutum* is more clearly visible in a side-by-side comparison of the synteny plots of Pima-S6 vs. TM-1 and Pima-S6 vs. Pima 3–79 HGS (Supplementary Fig. 4a, b). Structural variations in chromosomes A11, A12, A13, D05, D06 and D12 are common to both plots, indicating that the *G. barbadense* Pima 3–79 HGS assembly has a chromosome structure that is more similar to that of *G. hirsutum* TM-1, a genome from a sister yet different species, than that of Pima-S6, a genome from the same species. Furthermore, some chromosomal structural variations are only visible in either the Pima 3–79 HGS plot, e.g. the inversions in chromosome A05, or the TM-1 plot, e.g. the inversions in chromosome A03, suggesting that Pima 3–79 HGS scaffolding was not completely influenced by *G. hirsutum*. To further emphasize the influence of *G. hirsutum* on Pima 3–79 HGS chromosome structure, we re-scaffolded the 4,748 unplaced contigs using the RagTag pipeline, but this time using TM-1 genome as the reference. The resulting assembly was named Pima 3–79 RagTag-Gh. The synteny plot between Pima-S6 and Pima 3–79 RagTag-Gh recapitulates all the major chromosomal rearrangements present in the Pima-S6 vs. Pima 3–79 HGS plot (Supplementary Fig. 4d).

**Fig. 3:**
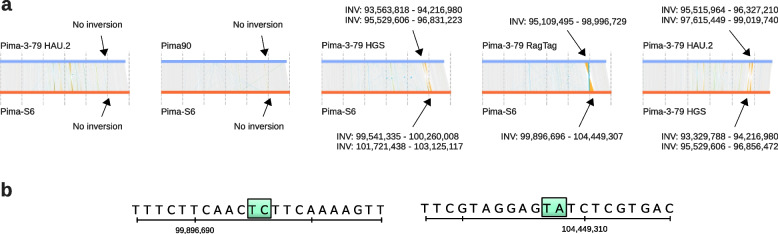
Chromosome A01 Pima-S6 vs. Pima 3–79 synteny plots. (**a**) Chromosome A01 synteny plots. A synteny analysis was carried out between chromosome A01 of the different assemblies indicated on the left side of each plot. Inversion (Inv) coordinates are indicated above and below each plot. Vertical lines represent syntenic (grey), inverted (orange), or duplicated (blue) regions. Note that there is no inversion in the Pima 3–79 HAU.2 vs. Pima-S6 plot at the coordinates where an inversion is present in the Pima 3–79 vs. Pima-S6, Pima 3–79 RagTag vs. Pima-S6 or Pima 3–79 HAU.2 vs. Pima 3–79 HGS plots. A close-up on the Pima-S6 inversion boundaries (**b**) in the Pima-S6 vs. Pima 3–79 RagTag plot showed that the inversion starts and ends at unambiguously assembled regions

Re-scaffolding of the Pima 3–79 HGS unplaced contigs eliminated all major chromosomal structural variations that were visible in the Pima-S6 vs Pima 3–79 HGS plot (Supplementary Fig. 4b), and the resulting Pima-S6 vs Pima 3–79 RagTag plot in Supplementary Fig. 4c indicates that the only major difference between the Pima-S6 and Pima 3–79 assemblies is a ~ 4 Mbp inversion in chromosome A01. Yet this inversion varies across Pima comparisons (Fig. 3a); it is absent in the Pima-S6 vs Pima 3–79 HAU.2 and Pima-S6 vs Pima90 plots; it is present as two shorter inversions in the Pima-S6 vs Pima 3–79 HGS plot; it is a single ~ 4 Mbp inversion in the Pima-S6 vs Pima 3–79 RagTag plot; and it is present as two shorter inversions in a Pima 3–79 HGS vs Pima 3–79 HAU.2 plot. These findings prompted us to ask which Pima chromosomal structural variations are “real” and which are due to contig or scaffold misplacement during pseudo-chromosome reconstruction.

A closer look on chromosome A01 indicated that this(ese) inversion(s) appear to originate at the assembly step, and not during our re-scaffolding process. The Pima-S6 vs. Pima 3–79 RagTag chromosome A01 inversion is fully contained within Pima 3–79 contig ML705862.1 (the inversion is located at coordinates 95,109,495 to 98,996,729 and the contig is at coordinates 94,907,062 to 99,420,900). The chromosome A01 inversion in Pima-S6 is also fully contained within Pima-S6 scaffold463 (inversion at coordinates 99,896,696 to 104,449,307, scaffold at coordinates 99,775,320 to 120,119,894), and the Pima-S6 inversion boundaries traverse unambiguously assembled regions (Fig. 3b). This chromosome A01 inversion therefore originated at the contig/scaffold sequence level and indicates that the Pima 3–79 HGS contig ML705862.1 was assembled as a fully or partially inverted sequence relative to Pima-S6 or Pima 3–79 HAU.2. Because of the inability to obtain the unplaced contigs of the Pima90 or Hai7124 assembly (not publicly available), at this point we can conclude that the only possible major chromosomal rearrangement within *G. barbadense* Pima cotton genomes is in chromosome A01.

#### Comparison of annotated proteins across *Gossypium* assemblies

After examining the differences between the Pima-S6 assembly and other *Gossypium* assemblies at the chromosome sequence level, we proceeded to compare the annotated proteins across them. We compared the Pima-S6 protein sequences to Pima 3–79 RagTag (which we believe to be a more accurate representation of the Pima 3–79 HGS genome assembly, see Orthogroups section in Materials and Methods), Pima 3–79 HAU.2, Hai7124 (all AD_2_ genomes) and TM-1 (AD_1_ genome) proteins, and to the annotated proteins from three additional tetraploid genome assemblies, *G. tomentosum* (AD_3_), *G. mustelinum* (AD_4_), *G. darwinii* (AD_5_) and three diploid genome assemblies *G. herbaceum* (A_1_), *G. arboreum* (A_2_) and *G. raimondii* (D_5_). We *de novo* annotated the Pima 3–79 RagTag assembly using our pipeline, resulting in 74,761 genes, of which 98.9% (73,941 genes) are located on the 26 chromosomes. The Pima 3–79 RagTag annotation had better protein BUSCO scores than the original Pima 3–79 HGS annotation, with 96.9% (subgenome A) and 97.4% (subgenome D) of complete eudicots_odb10 dataset BUSCOs vs. 94.6% and 96.2% for the Pima 3–79 HGS proteins (Fig. 1).

**Fig. 4:**
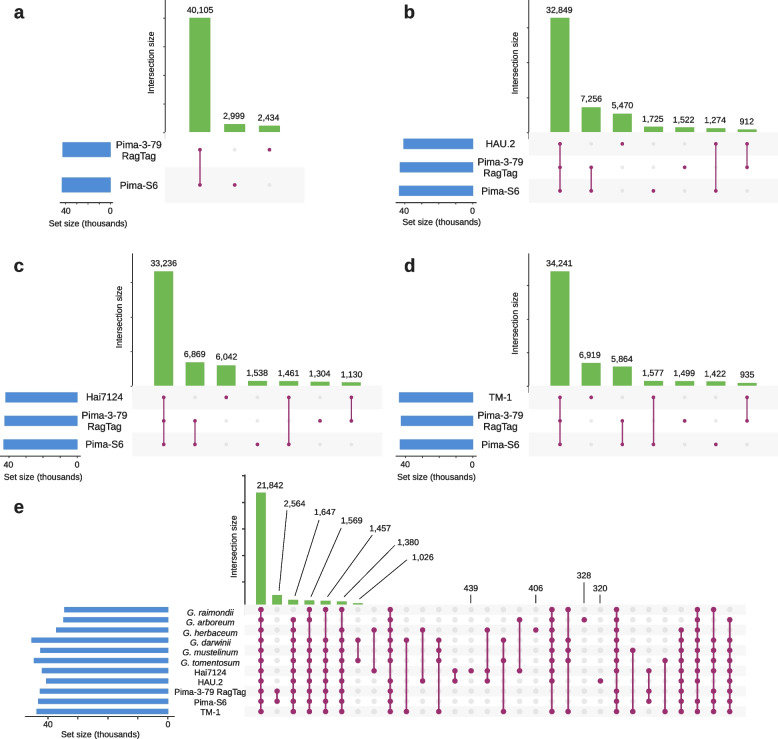
Number of orthogroups shared between *Gossypium* genome assemblies. In these UpSet^63^ plots, set size indicates the number of orthogroups for each species, and intersection size indicates the number of orthogroups in each of the intersections shown below the corresponding bar. A total of 62,760 phylogenetic hierarchichal orthogroups were identified from the annotated proteins of G. hirsutum TM-1, *G. barbadense* Pima-S6, *G. barbadense* Pima 3–79 RagTag, *G. barbadense* Pima 3–79 HAU.2, *G. barbadense* Hai7124, *G. tomentosum*, *G. mustelinum*, *G. darwinii*, *G. herbaceum*, *G. arboreum* and *G. raimondii*. (a) Number of orthogroups shared between Pima-S6 and Pima 3–79 RagTag. Over 90% of the orthogroups are shared between both assemblies. (b) Number of orthogroups shared between three Pima assemblies, Pima-S6, Pima 3–79 RagTag and Pima 3–79 HAU.2. A majority of orthogroups are shared between the three assemblies. Pima-S6 and Pima 3–79 RagTag also share an important number of orthogroups that are absent in HAU.2, and vice versa. This same pattern is observed in (c) and (d), with a majority of orthogroups shared between the three species plotted, an important number of orthogroups only shared by Pima-S6 and Pima 3–79 RagTag, and an important number of orthogroups exclusive of the third assembly. (e) Number of orthogroups shared across all eleven assemblies. Only the first 30 most abundant intersections were plotted, with the last intersection shown having 272 orthogroups. 222 orthogroups are exclusive of Pima-S6, 265 of Pima 3–79 RagTag and 66 of TM-1. Note that the second most abundant intersection is that of orthogroups exclusive of Pima-S6 and Pima 3–79 RagTag.

We identified 62,760 phylogenetic hierarchical orthogroups across all 11 assemblies. Diploid assemblies were represented in 37,090 (*G. herbaceum*; A_1_), 34,706 (*G. arboreum*; A_2_) and 35,382 (*G. raimondii*; D_5_) orthogroups; while tetraploid assemblies were represented in 43,672 (*G. hirsutum*; AD_1_), 43,104 (*G. barbadense* Pim-S6; AD_2_), 40,505 (*G. barbadense* Pima 3–79 HAU.2; AD_2_), 42,539 (*G. barbadense* Pima 3–79 RagTag; AD_2_), 41,869 (*G. barbadense* Hai7124; AD_2_), 44,548 (*G. tomentosum*; AD_3_), 42,405 (*G. mustelinum*; AD_4_) and 45,335 (*G. darwinii*; AD_5_) orthogroups. A comparison between Pima-S6 and Pima 3–79 RagTag showed that 40,105 orthogroups contained proteins from both genomes, while 2,999 orthogroups contained Pima-S6 proteins without a Pima 3–79 RagTag ortholog, and 2,434 orthogroups contained Pima 3–79 RagTag proteins without a Pima-S6 ortholog (Fig. 4a). This indicates that, while sharing over 90% of their orthogroups, there are still proteins from one genome that have no ortholog in the other genome. A comparison between Pima-S6, Pima 3–79 RagTag and either Pima 3–79 HAU.2 or Hai7124 (Fig. 4b, c) showed that over 33,000 orthogroups were shared between the three assemblies, around 7,000 orthogroups contain Pima-S6 and Pima 3–79 RagTag proteins without a Pima 3–79 HAU.2 or Hai7124 ortholog, and 5,470 (13% of the 40,505 HAU.2 total) orthogroups contained Pima 3–79 HAU.2 proteins and 6,042 (14% of the 41,869 Hai7124 total) orthogroups contained Hai7124 proteins, without a Pima-S6 nor a Pima 3–79 RagTag ortholog.

When Pima-S6 and Pima 3–79 RagTag were compared to TM-1, again a majority of orthogroups contained proteins from the three species, while 5,864 orthogroups contained Pima S-6 and Pima 3–79 RagTag proteins without a TM-1 ortholog, and 6,919 contained TM-1 proteins without a Pima-S6 or Pima 3–79 RagTag ortholog. Finally, the comparison across all 11 *Gossypium* genomes (Fig. 4e) showed that 1) a majority of orthogroups are shared between all genomes; 3) 2,564 orthogroups, the second most abundant intersection in Fig. 4e, contain proteins exclusive to Pima-S6 and Pima 3–79 RagTag; 3) the next four most abundant intersections are between all tetraploid species and all diploid species except either *G. raimondii*, *G. herbaceum*, *G. herbaceum* and *G. arboreum*, or *G. arboreum*, meaning that the majority of ortohogroups are shared between all species or all species except one or two diploid species; and 4) there are genome-specific orthogroups for each of the 11 assemblies, with 222 Pima-S6-specific, 265 Pima 3–79 RagTag-specific and 66 TM-1-specific orthogroups.

## GO categories enrichment analysis of Pima-S6, Pima 3–79 RagTag and TM-1-specific proteins

**Fig. 5:**
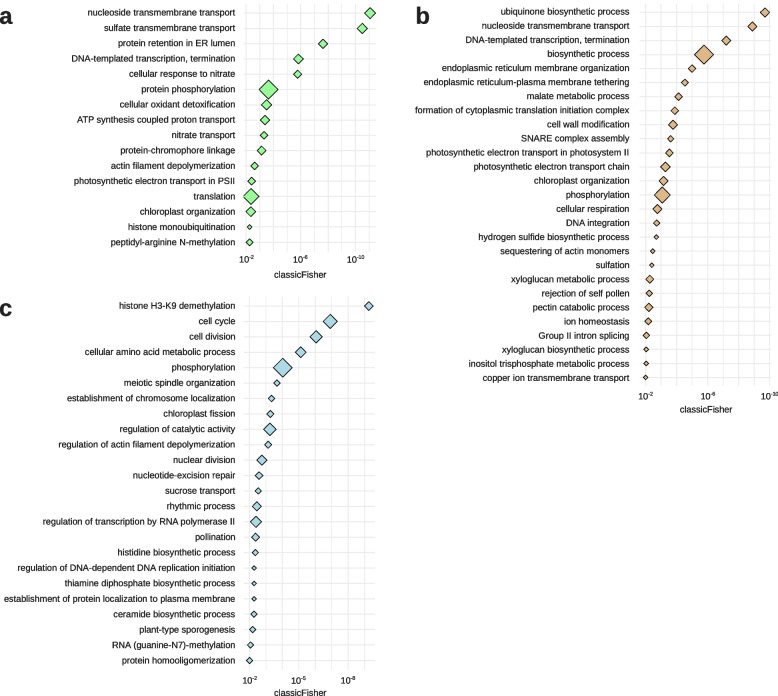
Enriched GO biological process categories in Pima-S6, Pima 3–79 RagTag and TM-1-specific orthogroups. Protein ids from Pima-S6 (a), Pima 3–79 RagTag (b) and TM-1 (c) -specific orthogroups were recovered, and the corresponding lists of gene IDs were analyzed for GO biological process categories enrichment with topGO. Dots represent GO biological process categories, and dot size is proportional to the number of annotated genes in the category. X-axis scale indicates the classicFisher value from the topGO analysis. Only categories with a classicFisher value of 0.01 or lower are presented. The list of Pima-S6-specific proteins is enriched in membrane transport and photosynthesis categories, while the list of Pima 3–79 RagTag specific proteins is enriched in cell wall metabolism categories and the list of TM-1-specific proteins is enriched in cell division categories

To identify the biological processes in which species and cultivar-specific genes contribute, we performed a GO biological process categories enrichment analysis of the lists of genes coding Pima-S6, Pima 3–79 RagTag, and TM-1-specific proteins using a *de novo* functional annotation for these three genome assemblies (Fig. 5). The list of Pima-S6-specific proteins is enriched (pvalue < = 0.01) in transmembrane transport and photosynthesis categories (Fig. 5a), while the list of Pima 3–79 RagTag-specific proteins is enriched in cell wall metabolism categories (Fig. 5b), and the list of TM-1-specific proteins is enriched in cell division categories (Fig. 5c). To identify *G. barbadense*-specific enriched categories, we retrieved the list of genes present in Pima-S6 and/or Pima 3–79 RagTag, two genomes that we annotated using our pipeline, but absent in non-*G. barbadense* genomes. This *G. barbadense*-specific gene list is enriched mainly in photosynthesis, ATP production and Carbon fixation categories (pvalue < = 0.01), however, at p-values between 0.01 and 0.05 we found several cell wall-related categories: GO:0045490, pectin catabolic process; GO:0045227, capsule polysaccharide biosynthetic process; GO:0042732, D-xylose metabolic process; GO:0033358, UDP-L-arabinose biosynthetic process; GO:0033320, UDP-D-xylose biosynthetic process, and GO:1,901,141, regulation of lignin biosynthetic process (Supplementary Data 1). The Enzyme Commission (E.C.) numbers assigned by the PlantCyc E2P2 pipeline to the genes in these categories corresponded to D-xylose and D-arabinose metabolism (E.C. 4.1.1.35, UDP-glucuronate decarboxylase; E.C. 5.3.1.5, xylose isomerase; E.C. 5.1.3.2, UDP-glucose 4-epimerase; E.C. 5.1.3.5, UDP-arabinose 4-epimerase) and pectin metabolism (E.C. 3.1.1.11, pectinesterase; E.C. 4.2.2.2, pectate lyase) reactions. No E.C. numbers were assigned to the four genes in the GO:1901141 regulation of lignin biosynthetic process category (Supplementary Data 1). These results suggest that the metabolism of non-cellulosic primary cell wall components is an important feature of Pima-S6 and Pima 3–79.

## Discussion

### What is the “real” structure of *G. barbadense* chromosomes?

In this study we report a genome assembly of *Gossypium barbadense* L. of Pima-S6, the source of Fusarium wilt [*Fusarium oxysporum* f. sp. *vasinfectum* (FOV)] race 4 (FOV4) resistance to the first commercial Pima varieties and public germplasm releases in the USA [11, 32]. An assembly of 2,301,422,177 bp was obtained. BUSCO statistics and repeats content showed that our Pima-S6 genome is comparable to previously reported Gossypium genome assemblies, and BUSCO statistics suggest that our annotation is slightly more complete than these other Gossypium genomes. When compared at the chromosome and protein sequence-level to other *Gossypium* genomes, differences appeared regarding chromosomal structural variations, i.e., synteny, inversions, translocations, and duplications, and the number of protein orthogroups shared between *Gossypium* genomes.

The synteny analysis between Pima-S6 and *G. hirsutum* TM-1 showed that both genomes have important differences in chromosome structure. The end points of the major inversions fell inside non-ambiguous sequences, suggesting they were not assembly artifacts. But when we compared our Pima-S6 assembly to other *G. barbadense* assemblies, questions about the “real” chromosome structure of *G. barbadense* genomes arose. Since we always used Pima-S6 as the query genome in all synteny analyses of Fig. 2, we expected structural variations to be mostly consistent across comparisons. Yet this is not what we observed. The Pima-S6 vs. HAU.2 is more like the Pima-S6 vs. Pima90 plot than the Pima-S6 vs. Pima-3-79 HGS plot, when the HAU.2 and Pima-3-79 HGS assemblies originate from the same cultivar. Two major inversions in chromosomes A09 and D05 are common to the Pima-S6 vs. HAU.2 and Pima-S6 vs. Pima90 plots, and these inversions correspond to scaffolds of the Pima-S6 assembly. On the other hand, the inversion in chromosome A13 appears to be common to the comparisons of Pima-S6 vs. Pima-3-79 HAU.2, Pima-3-79 HGS and Pima90. Note that Pima-3-79 HAU.2 and Pima90 pseudo-chromosome reconstruction did not involve any alignment to a previous Gossypium genome. These results suggest that (1) the use of the TM-1 UTX v2.1 assembly as a scaffolding reference for Pima-S6 pseudo-chromosomes reconstruction introduced three major inversions in chromosomes A09, A13 and D05; (2) the HAU.2 and Pima90 chromosome structures would be closer to the “real” *G. barbadense* chromosome structure, with the remaining differences between these two plots reflecting the differences between the Pima-3-79 and Pima90 cultivars; (3) the pseudo-chromosome reconstruction of the Pima-3-79 HGS assembly inadvertently misplaced contigs; and (4) if the Pima-3-79 HGS assembly has misplaced contigs, then the presence of common structural variations between the Pima-S6 vs. Pima-3-79 HGS and Pima-S6 vs. Hai7124 plots would cast doubt on the structure of some Hai7124 pseudo-chromosomes.

The chromosomal structural differences we observed are unlikely to originate from crossing or introgression between cultivars or between species. Pima-S6, Pima 3–79 and Pima 90 all shared original common breeding pools. In addition, Pima cotton has traditionally been more prone to rank growth compared with Upland (*G. hirsutum* L.) cotton, and is not commonly grown in environments where growing season length is limiting. Consequently, Pima cotton is not predominantly grown in Texas. Pima also differs from Upland cotton in numerous traits. Differences in yield, fiber quality, and growth habit between these two species have been observed in other states, such as Arizona and California. Differences in production regions, flower morphology combined with pollen production or stigma reception (late for Pima) have been kept domestication introgression between Pima and Upland low to nonexistent. In addition, Pima cotton is highly valued in the premium textile market because of its superior fiber fineness, length, and strength qualities. Historically, Pima cotton have been used to introgress this fiber quality into Upland cotton, which is why Upland cottons contain more introgression(s) from Pima, and not the other way around.

### Structural differences between *G. barbadense* genomes likely originate at the pseudo-chromosome reconstruction step

Synteny analyses showed that our Pima-S6 assembly has at least three major inversions, in chromosomes A09, A13 and D05, when compared to Pima-3-79 HAU.2 and Pima90, suggesting they are artifacts introduced when we used the TM-1 assembly as the scaffolding reference. However, all other major structural variations between Pima-S6 and Pima-3-79 HAU.2, Pima-3-79 HGS and Pima90 appear to be present in one comparison and absent in the other two. Furthermore, a re-scaffolding of the Pima-3-79 HGS unplaced contigs resulted in the Pima-3-79 RagTag re-assembly that has no major chromosomal structural variations, except for an inversion in chromosome A01, an inversion that is not present in all *G. barbadense* comparisons (Fig. 3). This indicates that, at the contig level, Pima-S6 and Pima-3-79 are essentially syntenic, and from the synteny plots we can further infer that Pima90 is also essentially syntenic to these two assemblies.

The synteny we observed between Pima-S6 and Pima-3-79 RagTag, and the similarities between the Pima-S6 vs. Pima-3-79 HAU.2 and Pima-S6 vs. Pima90 synteny plots would imply that the differences we observed between the Pima-S6, Pima-3-79 and Pima90 genomes originated at the pseudo-chromosome reconstruction step. Pseudo-chromosomes reconstruction of Pima-3-79 HGS and Pima90 included the use of Hi-C data. The almost complete synteny between Pima-S6 and Pima-3-79 RagTag suggested that Pima-S6, Pima-3-79 and Pima90 are similar enough at the nucleotide sequence level, and therefore it would be possible to align Hi-C data from one cultivar onto the other to validate or invalidate the presence of eventual inversions. Hi-C reads for Pima90 are publicly available, and an analysis of this data versus the Pima-S6 and Pima-3-79 HGS genomes did confirm the presence of the three inversions in chromosomes A09, A13 and D05 of Pima-S6, and the absence of the A13 inversion in Pima-3-79 HGS (Supplementary Fig. 6). A combination of in-depth synteny and Hi-C data analyses from Pima-S6, Pima-3-79, Pima90 and Hai7124 between these same assemblies, and between assemblies obtained from the re-scaffolding of unplaced contigs from one assembly using the others as reference, could help us elucidate the “real” structure of *G. barbadense* chromosomes. Unfortunately, the unplaced contigs for Pima-3-79 HAU.2 and Pima90 are not publicly available. Hi-C data is publicly available for Hai7124, but not the unplaced contigs. Hi-C data for Pima-S6 is presently unavailable, and Hi-C data for Pima-3-79 HGS is also unavailable, or the files were mislabeled during upload to the sequencing reads databases.

### *Gossypium* genomes probably have more than 75,000 genes

Genome assembly is followed by genome annotation, which our orthogroups analyses showed that is also an important technical step (Fig. 4 and Supplementary Fig. 7, see Materials and Methods). Our genome annotation pipeline differs from those used by Hu and colleagues (2019) for *G. barbadense* Hai7124 [15], Wang and colleagues (2019) for Pima 3–79 HAU.2 [20] or Chen and colleagues (2020) for Pima 3–79 (HGS [5]). These three research teams used in-house strategies that leveraged different combinations of sequencing data and gene prediction software. We opted for a more straightforward and reproducible approach by using MAKER-P for Pima-S6 and Pima 3–79 RagTag annotation, followed by a conservative approach that filtered all genes identified by MAKER-P that did not show homology to known proteins. The pre-homology filter results from MAKER-P indicated the presence of ~ 88,000 genes for Pima-S6 and Pima 3–79 RagTag, which were reduced to ~ 75,000 genes after filtering. BUSCO scores are identical between the 88,000 and 75,000 proteins derived from these annotations (2,311 complete, 4 fragmented, 11 missing BUSCOs for the 2,326 proteins eudicots_odb10 dataset), although this is not surprising since BUSCO is based on homology to known proteins. This conservative filtering approach used by us, but also by others, for example Chen and colleagues (2020), could potentially lead to discarding loci without protein-coding regions. Long non-coding RNA loci are an example of loci without protein-coding potential, and in fact 8,514 long noncoding RNAs have been identified in *G. hirsutum* [33] and 8,113 in *G. arboreum* [34] to cite two reports. In addition, 1,965 Pima-S6 and 2,163 Pima 3–79 RagTag genes whose proteins lack homology to know proteins had evidence of expression in an RNA-seq dataset obtained from roots and leaves, and 5,272 Pima 3–79 RagTag genes had evidence of expression in a more comprehensive RNA-seq dataset from BioProject PRJNA266265. Although these numbers are still below the ~ 13,000 genes difference between our pre- and post-filtered annotations, they do support the possibility that cotton genomes contain more transcribed functional loci than the ~ 75,000 usually reported, and in fact Ma and colleagues (2021) reported 79,613 protein-coding genes for Pima90. Cotton genome annotations could be further refined by adding to annotation strategies the use of stranded non-polyA short reads and/or long reads RNA sequencing libraries, which are still currently unavailable for Pima-S6.

### There are cultivar-specific *Gossypium* proteins

An orthogroups analysis of 11 *Gossypium* species showed that there are Pima-S6 proteins without an ortholog in Pima 3–79 RagTag and vice versa, and that there are in fact species and cultivar-specific proteins in all 11 *Gossypium* genomes. Chen and colleagues (2020) performed an orthogroups analysis using five *Gossypium* species (*G. hirsutum*, *G. barbadense*, *G. tomentosum*, *G. mustelinum* and *G. darwinii*), and did not find any assembly species-specific orthogroups. This striking difference with our results could be due to the use of different versions of the Orthofinder software that was used in both analyses, or the fact that the five genomes analyzed by Chen and colleagues (2020) were annotated using the same pipeline. Orthofinder was recently improved, and now outputs phylogenetic hierarchical orthogroups, which the authors qualify as more accurate than the orthogroups generated by previous versions. As for genome annotation pipelines, the increase in the number of shared orthogroups in the Pima-S6 vs. Pima 3–79 RagTag comparison relative to the Pima-S6 vs. Pima 3–79 HGS comparison (Fig. 5a vs. Supplemental Fig. 7a) suggests that the number of species and cultivar-specific orthogroups across *Gossypium* assemblies could eventually be reduced if all 11 assemblies were first annotated using the same pipeline. Still, the existence of proteins specific to Pima-S6 and Pima 3–79 RagTag, two closely related cultivars whose genomes we annotated using the same pipeline, does support existence of species and cultivar-specific proteins.

### Species and cultivar-specific proteins have distinct biological roles

We performed a GO biological process categories enrichment analysis of the Pima-S6, Pima-3-79 RagTag and TM-1 proteins without an ortholog in any of the other 10 *Gossypium* assemblies analyzed. These proteins are distinctive of a molecular functional process of each assembly and should provide information as to the molecular functions that contribute to the phenotypic particularities of each cotton species and cultivar. Each protein-coding gene list was enriched for a different set of biological process categories. The Upland TM-1 protein list was enriched in categories related to the cell division processes, whereas the Pima 3–79 RagTag list was enriched in a set of primary cell wall metabolism categories, such as xyloglucan biosynthesis and pectin catabolism, as well as endoplasmic reticulum and SNARE complex assembly categories, which could reflect membrane fusion events involved in cell wall polymers export to the apoplast. Pima-S6 was not enriched in any cell wall-related categories but was enriched in a set of membrane transport and photosynthesis categories. The list of proteins that are present in either Pima-S6, Pima 3–79 RagTag or both without an ortholog in non-*G. barbadense* species was enriched in categories involving non-cellulosic primary wall metabolism enzymes. These categories would suggest that hemicellulose and/or pectin metabolism are important for the longer and higher quality fibers, i.e., primary cell walls, characteristic of Pima cotton [35, 36]. Our orthogroup analyses suggest that presence/absence variation exists between *G. barbadense* and *G. hirsutum*, but also between two closely related *G. barbadense* cultivars. Research is ongoing to further analyze where and when these species and cultivar-specific genes are expressed to better evaluate their contribution to the distinct phenotypes of Pima-S6, Pima 3–79 and TM-1 plants and organs. This information will help to elucidate genes for important traits such as FOV4 resistance and fiber improvement and assist in the breeding processes of future breeding programs.

## Conclusion

In this work we have presented a novel genome assembly for the *G. barbadense* Pima-S6 cultivar. Comparison of this assembly at the chromosome and protein sequence levels to other *Gossypium* species showed that the results are dependent on the methodologies used to obtain such assemblies/sequences. Our work emphasized the need for some form of homologation of sequence assembly, pseudo-chromosome reconstruction, and genome annotation to be able to do more precise comparative genomics.

The development of this Pima-S6 genome, a new cotton genomic resource, offers new insights into *G. barbadense* chromosomes structure. It will facilitate the identification of genes and alleles important for crop improvement, and the identification of recombination events and selection signatures important for yield, fiber quality, and disease resistance such as resistance Fusarium wilt [*Fusarium oxysporum* f. sp. *vasinfectum* race 4 (FOV4)], speeding future breeding processes.

## Methods

### Pima-S6 source

The original Pima S-6 source was released in 1984 [9] as a F_4_ selection from a cross of two experimental lines, 5934-23-2-6 and 5903-98-4-4. At the time of release, the major advantages of Pima S-6 were early maturity and high yield. We now know that Pima S-6 also possesses a major gene(s) for FOV4 resistance [10–12]. After being identified as a valuable source of FOV4 resistance and subjected to several cycles of field evaluations to increase its uniformity and the level of FOV4-resistance, this new selection resistance source was named “Pima-S6”. This source has been used in previous reported FOV4-related studies [10–13] and is the one reported herein as the entry used for whole genome sequencing. Seeds from the cotton germplasm collection from the USDA-ARS cotton breeding program were sown and grown at the USDA-ARS, Plant Stress and Germplasm Development Research Unit glasshouse in Lubbock, TX, USA.

### Genome assembly

High molecular weight DNA (HMW-DNA) of *G. barbadense* ‘Pima-S6’ was extracted from isolated nuclei and used to construct three different sequencing libraries. The genome was sequenced using paired-end (PE) and mate-pair (MP) libraries, with additional linked-reads sequencing libraries (10X genomics™ Chromium™; Supplementary Table 4). One shotgun library (160x) was made using DNA fragments of ~ 440 bp long as templates with no PCR amplification to produce ‘stitched’ reads of approximately 250 to 490 bp in length. To increase sequence diversity and genome coverage, a separate MP library (58x) was constructed with 2–5 Kbp long inserts using the Illumina Nextera Mate-Pair Sample Preparation Kit (Illumina, San Diego, CA). We obtained a total of ~ 504 Gb of sequencing data, equivalent to 219x genomic coverage based on an estimated genome size of 2.3 Gb. In addition, DNA fragments longer than 50 kb were used to construct a Gemcode library (82x coverage) using the Chromium instrument (10X Genomics, Pleasanton, CA). Pima-S6 genome assembly was conducted using the DeNovoMAGIC^™^ software platform (NRGene, Nes Ziona, Isreal). DeNovoMAGIC^™^ is a DeBruijn-graph-based assembler, designed to efficiently extract the underlying information in the raw reads to solve the complexity of the DeBruijn graph due to genome polyploidy, heterozygosity, and repetitiveness [15, 37–40]. The additional raw Chromium 10X data was used to phase polyploidy/heterozygosity, support scaffolds validation, and further elongate phased scaffolds. Pseudo-chromosomes were constructed based on their anchoring to a provided reference genome. As an initial step, the process detects suspected chimeric scaffolds and breaks them. Chimeric scaffolds are defined as scaffolds that have more than one significant mapping location in the reference genome (different chromosomes or distant locations within the same chromosome). Scaffolds which were split are supplemented with a suffix of “- <number>” to indicate the number of parts that the scaffold was split to. Next, scaffolds were mapped to the reference genome (TM-1 UTX_v2.1 [5]), and exact-match seeds larger than 64 bp were identified. For each scaffold, the maximal monotonic paths of exact-match seeds was efficiently calculated. Next, the optimal matching between scaffolds to the reference genome was heuristically approximated. This was done by iterating all max-monotonic-paths, starting with the best path and mapping scaffolds to vacant locations on the chromosome. The quality of a max-monotonic path was set by multiple factors such as homology & distribution of mapping, per scaffold and then relatively to the rest of the scaffolds. The order of scaffolds on the chromosomes was determined by the median of the mapped max-monotonic path. In each of those iterations the best mapped scaffolds per location on the reference genome were selected, while leaving the lower scored mappings for the following iterations.

### Pima-S6 chromosome naming

Chromosomes in our raw Pima-S6 assembly fasta file were named 1 to 26. We aligned the 26 chromosomes of our Pima-S6 assembly versus the 26 chromosomes of the *G. hirsutum* ‘TM-1’ UTX v2.1 or *G. barbadense* Pima 3–79 HGS [5] assemblies available at Phytozome [41] using minimap2 (v2.18) [42] with the parameter -x asm5. PAF output files were uploaded to a local instance of D-Genies [43], and the resulting dot plots (Supplementary Fig. 8) were used to identify homologous chromosomes and rename our Pima-S6 chromosomes accordingly.

### Genome annotation

Genome assemblies were annotated through three rounds of MAKER-P (v3.01.03) [24, 25]. Round 1 used as EST evidence all nucleotide sequences marked as EST from a search for “*Gossypium barbadense*” in the NCBI Nucleotide database (39,608 sequences, accessed Dec 22nd, 2020) for *G. barbadense* assemblies, or NCBI EST sequences from a search for “*Gossypium hirsutum*” (337,811 sequences, accessed Nov 12th, 2021), as protein evidence a collection of *Gossypium hirsutum* (v2.1, available at Phytozome) [5], *Arabidopsis thaliana* (Araport11 annotation) [44], *Zea mays* (NAM-5.0 assembly available at maizegdb) [45, 46] and *Populus trichocarpa* (v4.1 available at Phytozome) [47] proteins, and as the repeats file the *de novo* repeat sequences obtained using RepeatModeler (v2.0.1) with RepeatMasker (v4.1.2-p1) [48]; with Dfam v3.3 [49] and RepBase (v20181026) [50], and an LTR_retriever (v2.9.0) [51] run of LTR_Finder_parallel [52, 53], and ltr_harvest [54] results. Round 2 used as input the maker gff3 file from round 1, a SNAP hmm file obtained from round 1 gene models, and the Augustus [55] gene models from a BUSCO [28] run following Daren Card’s method (https://darencard.net/blog/2017-05-16-maker-genome-annotation/, Augustus section) with the eudicots_odb10 dataset. Round 3 was run as round 2, using round 2 output files as inputs. Round 3 gff3 files were used to recreate the CDS sequences using a custom Perl script, which were translated to protein sequences using the transeq program from the EMBOSS suite (v6.6.0). Genome assembly and annotation completeness were evaluated using BUSCO (v5.1.3) and the embryophyta_obd10 and eudicots_odb10 datasets. Genes without homology to known proteins, that is without an Interproscan Pfam match (evalue lower than 1^–10^), without a GO annotation (see Genome functional annotation section below) or without a BUSCO match vs. the eudicots_odb10 dataset were discarded from the gff3 file, and CDS and protein sequences were recreated and re-evaluated for completeness using BUSCO and the embryophyta_odb10 and eudicots_odb10 dataset.

### Genomic repeats annotation

Genomic long terminal repeat family’s content was determined using RepeatMasker (v4.1.2-p1) and our classified *de novo* repeats file for the A and D genomes, separately. LTR retrotransposon families were identified using the Domain based ANnotation of Transposable Elements (DANTE) tool from RepeatExplorer and the REXdb Viridiplantae v3.0 database [56]. The DANTE output file was filtered on the RepeatExplorer Galaxy server at https://repeatexplorer-elixir.cerit-sc.cz/ using default parameters.

### Genome functional annotation

Gene Ontology annotations for *G. barbadense* ‘Pima-S6’, *G. barbadense* ‘Pima 3–79 RagTag’ (our re-scaffolding of the *G. barbadense* ‘Pima 3–79’ HGS unplaced contigs) and *G. hirsutum* ‘TM-1’ UTX v2.1 [5] assemblies were assigned using a simplified version of the MAIZE-gamer pipeline [57]. Briefly, annotations were assigned as the GO annotations of the blastp reciprocal best hits versus Araport11 and UniProt Swiss-Prot proteins from nine plant species (*Glycine max*, *Oryza sativa* subsp. japonica, *Populus trichocarpa*, *Solanum lycopersicum*, *Sorghum bicolor*, *Vitis vinifera*, *Brachypodium distachyon*, *Physcomitrium patens*, and *Chlamydomonas reinhardtii*), the GO annotations from an Interproscan (v5.48.83) [58] analysis, and the GO annotations from the PANNZER2 [59] functional annotation webserver with a PPV value of at least 0.5. All these GO annotations were merged into a non-redundant gaf file.

### Identification of chromosome structural variations

Chromosome structural variations were identified using syri (v1.4) [60]. The 26 chromosomes of Gossypium assemblies were aligned using minimap2 (v2.18) [42] with the options -ax asm5 --eqx, and the resulting SAM output file was used as input for syri. Be advised that versions 2.19 and 2.20 of minimap2 results in syri outputs lacking many structural variations.

### Pima 3–79 HGS contigs re-scaffolding

The unplaced contigs for the *G. barbadense* Pima 3–79 HGS assembly [5] are publicly available under BioProject accession PRJNA516411. 4748 contigs, totaling 2,193,941,943 bp, were re-scaffolded using RagTag (v2.0.1) [31] in scaffold mode using the 26 chromosomes of our Pima-S6 assembly, or the *G. hirsutum* TM-1 assembly, as reference. RagTag was run with default parameters, which include the use of minimap2 (v2.18) as the aligner.

### RNA-seq data analysis

Total RNA from one leaf and one roots Pima-S6 or Pima 3–79 sample from 14 days old seedlings was isolated and sequenced as described previoulsy [61]. Pseudo-alignment and quantification of these leaves and roots samples (Pima-S6 and Pima 3–79) or the run accessions SRR1652328, SRR1652331, SRR1652333, SRR1652334, SRR1652335, SRR1652336, SRR1652337, SRR1652338, SRR1652339, SRR3098092, SRR3098093, SRR3098094, SRR3098095 and SRR3099009 from BioProject PRJNA266265 (Pima 3–79), were done using kallisto (v0.46.2) [62] and the ~ 88,000 transcripts from the unfiltered MAKER-P Pima-S6 or Pima 3–79 RagTag annotations as the index. Gene-level summarization of expression data was done with tximport [63]. Genes with an expression value of 1 TPM or higher in both leaves and root samples or in at least two PRJNA266265 samples were considered as being expressed.

### Orthogroups analyses

*Gossypium* protein orthogroups were identified using Orthofinder (v2.5.4) [64, 65] and the primary transcript proteins from 11 Gossypium genome assemblies: our *G. barbadense* Pima-S6 assembly, de novo annotated (AD_2_ genome), our *G. barbadense* Pima 3–79 Ragtag re-assembly, de novo annotated (AD_2_ genome), *G. barbadense* Pima 3–79 HAU.2 (AD_2_ genome, available from cottongen) [20], *G. barbadense* Hai7124 (AD_2_ genome, available from cottongen) [15], *G. hirsutum* TM-1 (AD_2_ genome, version 2.1 available from Phytozome) [5], *G. tomentosum* (AD_3_ genome, version 1.1 available from Phytozome) [5], *G. mustelinum* (AD_4_ genome, version 1.1 available from Phytozome) [5], *G. darwinii* (AD_5_ genome, version 1.1 available from Phytozome) [5]. For *G. barbadense* Pima 3–79 HAU.2 we used version .1 of the proteins as the primary transcript proteins. N0 phylogenetic hierarchical orthogroups were used for all comparisons.

The results presented in Fig. 4 showed that an important number of Pima-S6 and Pima 3–79 RagTag proteins are exclusive for these two assemblies, even when compared to other Pima 3–79 assemblies, other *G. barbadense* assemblies or other *Gossypium* assemblies. The existence of 10,414 orthogroups that contain Pima 3–79 HAU.2 proteins without a Pima 3–79 RagTag ortholog was particularly intriguing, since both genome assemblies were obtained from the same pedigree-cultivar. The differences between Pima 3–79 RagTag and Pima 3–79 HAU.2, which indicate differences at the protein sequence level, might be due to small genomic DNA sequence differences between Pima 3–79 seed-sources used for genome sequencing, differences in genome assembly pipelines, differences in genome annotation pipelines, or all of the above.

Since protein sequences are derived from genome annotations, it is likely that the latter process had a non-negligible influence on orthogroup analyses. To evaluate this, we re-ran our orthogroup analysis replacing the Pima 3–79 RagTag proteins with the proteins from the Pima 3–79 HGS annotation, and compared Pima-S6, Pima 3–79 HGS, Pima 3–79 HAU.2 and Hai7124, assemblies that were annotated each by a different research-team (Supplementary Fig. 7). In these new comparisons, fewer orthogroups are shared between Pima-S6 and Pima 3–79 HGS than between Pima-S6 and Pima-3-79 RagTag, and more orthogroups contain proteins from one assembly without an ortholog in the other assembly (Supplementary Fig. 7a); Pima 3–79 HAU.2 and Pima 3–79 HGS have an important number of proteins without an ortholog in the other assembly nor Pima-S6 (Supplemental Fig. 7b); and Pima 3–79 HGS has the highest number of orthogroups without orthologs in Pima-S6 or Hai7124 (Supplemental Fig. 7c). It is possible that the higher number of shared orthogroups between Pima-S6 and Pima 3–79 RagTag reflects a shared artifact or bias of our annotation pipeline. However, the ~ 75,000 Pima-S6 and Pima 3–79 RagTag proteins had improved BUSCO scores relative to the proteins from other *G. barbadense* assemblies or the TM-1 assembly, which would suggest that the Pima-S6 to Pima 3–79 RagTag comparison was more accurate than the Pima-S6 and Pima 3–79 HGS comparison. Note that our re-scaffolding of Pima 3–79 should have no incidence on protein sequence, as scaffolding to pseudo-chromosomes does not imply any genomic sequence alteration.

These orthogroup analyses, together with the improved BUSCO scores of the Pima 3–79 RagTag assembly, strongly suggest that the Pima 3–79 RagTag annotation is a more accurate representation of the Pima 3–79 gene content. These orthogroup analyses also showed that genome annotation pipelines play a significant role when genome assemblies are compared at the protein-coding gene level, and implies that protein sequences differ considerably from one annotation pipeline to the next. This would also imply that other analyses that involve protein sequence comparisons, for example protein coding sequence-based synteny analyses, will be equally affected.

### GO categories enrichment analysis

Protein ids from Pima-S6, Pima 3–79 RagTag and TM-1-specific orthogroups were retrieved and converted to their corresponding gene ids. The resulting gene lists were analyzed for GO Biological Process enrichment using topGO [66] with the weight01 graph method and fisher test statistic, our *de novo* functional annotations, and the list of mRNA-coding genes as the universe. Categories with a classicFisher value of 0.01 or lower were considered as significant.

### Hi-C analysis

Hi-C sequencing data for Pima90 [29], run accession SRR14506503 under BioProject PRJNA680449, was used as input for Juicer [67] versus either the Pima90 or Pima-S6 genomes. Hi-C contact plots were obtained from the resulting inter_30.hic files using Juicebox [68].

## Supplementary Information

**Additional file 1.**

**Additional file 2: Supplementary Figure 1.** Annotation Edit Distance scores of the Pima-S6 genome annotation. This histogram shows the distribution of Annotation Edit Distance (AED) scores, a measure of the goodness of fit of an annotation to the evidence supporting it, for the 75,419 genes in the final Pima-S6 annotation. From the MAKER/MAKER-P protocol (Campbell, M. S., Holt, C., Moore, B. and Yandell, M. 2014. Genome Annotation and Curation Using MAKER and MAKER-P. Curr. Protoc. Bioinform. 48:4.11.1- 4.11.39.; doi: 10.1002/0471250953.bi0411s48): “AED is a number between 0 and 1, with an AED of zero denoting perfectconcordance with the available evidence and a value of one indicating a complete absence of support for the annotated gene model. In other words, the AED score provides a measure of each annotated transcript’s congruency with its supporting evidence.” **Supplementary Figure 2.** Overview of leaf and root gene expression in Pima-S6 and Pima-3-79 RagTag. RNA from one leaves and one roots sample was isolated and sequenced, and gene expression was quantified. Bars represent the number of expressed genes (TPM > 1) in each species, organ and location on the A or D genome. **Supplementary Figure 3.a** LTR retrotransposon families distribution if four Gossypium assemblies, A genome. The 13 A genome chromosomes from Pima-S6, Pima-3-79 HGS, Hai7124 and TM-1 were analyzed using the Domain based ANnotation of Transposable Elements (DANTE) tool and the REXdb Viridiplantae v3.0 database. The output file was filtered on the Repeat Explorer Galaxy server at https://repeatexplorer-elixir.cerit-sc.cz/ using default parameters, and the number of sequencesper LTR retrotransposon family was plotted. **Supplementary Figure 3. b** LTR retrotransposon families distribution if four Gossypium assemblies, D genome. The 13 D genome chromosomes from Pima-S6, Pima-3-79 HGS, Hai7124 and TM-1 were analyzed using the Domain based ANnotation of Transposable Elements (DANTE) tool and the REXdb Viridiplantae v3.0 database. The output file was filtered on the RepeatExplorer Galaxy server at https://repeatexplorer-elixir.cerit-sc.cz/ using default parameters, and the number of sequences per LTR retrotransposon family was plotted. **Supplementary Figure 4.** Synteny plots between Pima-S6 and Pima-3-79 HGS, TM-1, Pima3-79 RagTag. and Pima3-79 RagTag-Gh In these synteny plots, the reference genome is represented by blue horizontal lines and the query genome by red horizontal lines. Vertical lines represent syntenic (grey), inverted (orange), translocated (green) and duplicated (blue) regions. Chromosome ids are indicated on the left side of the plots. The 26 chromosomes of our Pima-S6 assembly were aligned using minimap2 versus the 26 chromosomes of G. hirsutum TM-1 (a), Pima-3-79 HGS (b), Pima-3-79 HGS RagTag (our re-scaffolding of Pima-3-79 HGS using Pima-S6 as reference; c) or Pima-3-79 RagTag-Gh (our re-scaffolding of Pima-3-79 HGS using TM-1 as reference; d), and synteny analysis was carried out using syri. All major structural variations visible in the Pima-S6 vs TM-1 and Pima-S6 vs Pima-3-79 HGS plots are no longer present in the Pima-S6 vs Pima-3-79-RagTag plot, except for a ~4 Mbp inversion and ~0.5 Mbp duplication in chromosome A01, while the Pima-S6 vs Pima-3-79 RagTag-Gh plot recapitulates all the major chromosomal rearrangements from the Pima-S6 vs Pima-3-79 HGS plot. **Supplementary Figure 5**. Pima-S6 vs TM-1 chromosome A11 inversions. **a** Schematic representation of the chromosome A11 synteny. The TM-1 A11 chromosome is represented by a blue horizontal box and the scaffolds that form the Pima-S6 A11 chromosome are represented by red boxes. Scaffold ids are indicated below each box. The prefix “r” in a scaffold name indicates a scaffold placed in reverse orientation. Gaps between scaffolds represent the 100 bp N gaps introduced during chromosome reconstruction. The three major inversions (> 1 Mbp) are represented by orange hourglasses. For each inversion a zoom on the sequence at the inversion start and end is shown, with the exact inversion boundaries highlighted by green boxes. Inversion1's boundaries traverse unambiguously assembled regions. Inversion2 starts at an unambiguously assembled region and ends at the start of a 1,000 bp N stretch. Inversion3 starts at the beginning of scaffold1213-2 and ends at an unambiguously assembled region. **b** Detailed view of the inversion2 to inversion3 region. Inversion2 is followed by a 1,000 bp N stretch (represented by the letter N inside rscaffold116) and 535 bp of unambiguous sequence. These 535 bp are themselves an inversion-duplication (invdp) nested within inversion2 on the TM-1 side. (**c**) IGV screenshot of the rscaffold116 1,000 N region. Re-alignment of the mate-pair reads showed uniquely mapped reads (bowtie2 XS flag null and not duplicate filter) aligned to rscaffold116's 535 bp end with their mate thousands of bp upstream, confirming the linkage evidence across the 1,000 bp N stretch. **Supplementary Figure 5.** TM-1 vs Pima-S6 synteny plots for chromosomes A03, A12 and D12. All inversion boundaires, except the end of inversion1 on chromosome A03, traverse unambiguously assembled regions. The 3' end of inversion1 on chromosome A03 is followed by a 10 bp N stretch and a 1,532 bp inversion-duplication of unambiguously assembled sequence that aligns to a chromosome A11 region on the TM-1 side. **Supplementary Figure 6.** Hi-C plots of Pima90 Hi-C reads aligned to the Pima90, Pima-S6 and Pima-3-79 HGS genomes. In these plots, the blue color indicates a chromatin contact. The coordinate 1 of each chromosome is located at the top left corner. Only chromosomes A09, A13 and D05 are shown. In the Pima-S6 plot, the inversions in these three chromosomes are clearly visible (orange arrows). In the Pima-3-79 HGS plot, the inversions in chromosomes A09 and D05 are also clearly visible, but an inversion in chromosome A13 is not apparent. The Pima90 plot is shown as reference. **Supplementary Figure 7.** Number of orthogroups shared between Pima-S6, Pima-3-79 HGS, Pima-3-79 HAU.2 and Hai7124. In these UpSet plots, set size indicates the number of orthogroups for each species, and intersection size indicates the number of orthogroups in each of the intersections shown below the corresponding bar. A total of 75,263 orthogroups were identified from the annotated proteins of G. hirsutum 'TM-1' (genome AD1), G. barbadense 'Pima-S6' (AD2; Pima-S6), G. barbadense 'Pima-3-79 HGS' (AD2; Pima-3-79 HGS), G. barbadense 'Pima-3-79' HAU.2 (AD2; HAU.2), G. barbadense Hai7124 (AD2; Hai7124), G. tomentosum (AD3), G. mustelinum (AD4), G. darwinii (AD5), G. herbaceum (A1), G. arboreum (A2) and G. raimondii (D5). (a) Number of orthogroups shared between Pima-S6 and Pima-3-79 HGS. The number of shared orthogroups is lower than the number of shared orthogroups between Pima-S6 and Pima-3-79 RagTag in Figure 5a. (b) Number of orthogroups shared between three Pima assemblies, Pima-S6, Pima-3-79 HGS and Pima-3-79 HAU.2. Pima-3-79 HGS and Pima-3-79 HAU.2 have an important number of proteins without an ortholog in any of the other two assemblies. (c) Number of orthogroups shared between Pima-S6, Pima-3-79 HGS and Hai7124. Pima-3-79 HGS has the highest number of orthogroups without orthologs in Pima-S6 or Hai7124. **Supplementary Figure 8.** Dot plots of Pima-S6 vs Pima-3-79 HGS and TM-1 genome assemblies alignments. The 26 chromosomes of the indicated genome assemblies were aligned using minimap2, and the PAF alignment file was plotted using D-Genies. Pima-S6 chromosome ids were renamed to the AD nomenclature using the best matching chromosome in Pima-3-79 HGS and TM-1: chromosome 1 was renamed A01, chromosome 2 D01, chromosome 3 A02, chromosome 4 D02, and so on. **Supplementary Table 1.** Statistics for Pima-S6 and Pima-3-79 RNA-seq leaf and root RNA-seq samples. Supplementary Table 2.

## Data Availability

The Pima-S6 genome assembly, Pima-S6 and Pima 3–79 leaves and roots RNA-seq data are available under NCBI BioProject accession PRJNA798371. The Pima 3–79 RagTag assembly, Pima-S6 and Pima 3–79 RagTag unfiltered and filtered genome annotations, and Pima-S6, Pima 3–79 and TM-1 functional annotations are available at Cyverse. Correspondence and materials requests should be addressed to Mauricio Ulloa.
