# Supplementary material for: Assembly and annotation of the Gossypium barbadense L. ‘Pima-S6’ genome raise questions about the chromosome structure and gene content of Gossypium barbadense genomes
Source: BMC Genomics. 2023 Jan 10;24:11. doi: 10.1186/s12864-022-09102-6 (PMC9830710; doi:10.1186/s12864-022-09102-6)
Supplement: Supplementary file 2 — Additional file 2: Supplementary Figure 1. Annotation Edit Distance scores of the Pima-S6 genome annotation. This histogram shows the distribution of Annotation Edit Distance (AED) scores, a measure of the goodness of fit of an annotation to the evidence supporting it, for the 75,419 genes in the final Pima-S6 annotation. From the MAKER/MAKER-P protocol (Campbell, M. S., Holt, C., Moore, B. and Yandell, M. 2014. Genome Annotation and Curation Using MAKER and MAKER-P. Curr. Protoc. Bioinform. 48:4.11.1- 4.11.39.; doi: 10.1002/0471250953.bi0411s48): “AED is a number between 0 and 1, with an AED of zero denoting perfectconcordance with the available evidence and a value of one indicating a complete absence of support for the annotated gene model. In other words, the AED score provides a measure of each annotated transcript’s congruency with its supporting evidence.” Supplementary Figure 2. Overview of leaf and root gene expression in Pima-S6 and Pima-3-79 RagTag. RNA from one leaves and one roots sample was isolated and sequenced, and gene expression was quantified. Bars represent the number of expressed genes (TPM > 1) in each species, organ and location on the A or D genome. Supplementary Figure 3.a LTR retrotransposon families distribution if four Gossypium assemblies, A genome. The 13 A genome chromosomes from Pima-S6, Pima-3-79 HGS, Hai7124 and TM-1 were analyzed using the Domain based ANnotation of Transposable Elements (DANTE) tool and the REXdb Viridiplantae v3.0 database. The output file was filtered on the Repeat Explorer Galaxy server at https://repeatexplorer-elixir.cerit-sc.cz/ using default parameters, and the number of sequencesper LTR retrotransposon family was plotted. Supplementary Figure 3. b LTR retrotransposon families distribution if four Gossypium assemblies, D genome. The 13 D genome chromosomes from Pima-S6, Pima-3-79 HGS, Hai7124 and TM-1 were analyzed using the Domain based ANnotation of Transposable Elements (DANTE) tool and the REXdb Viridi [file 12864_2022_9102_MOESM2_ESM.pdf]

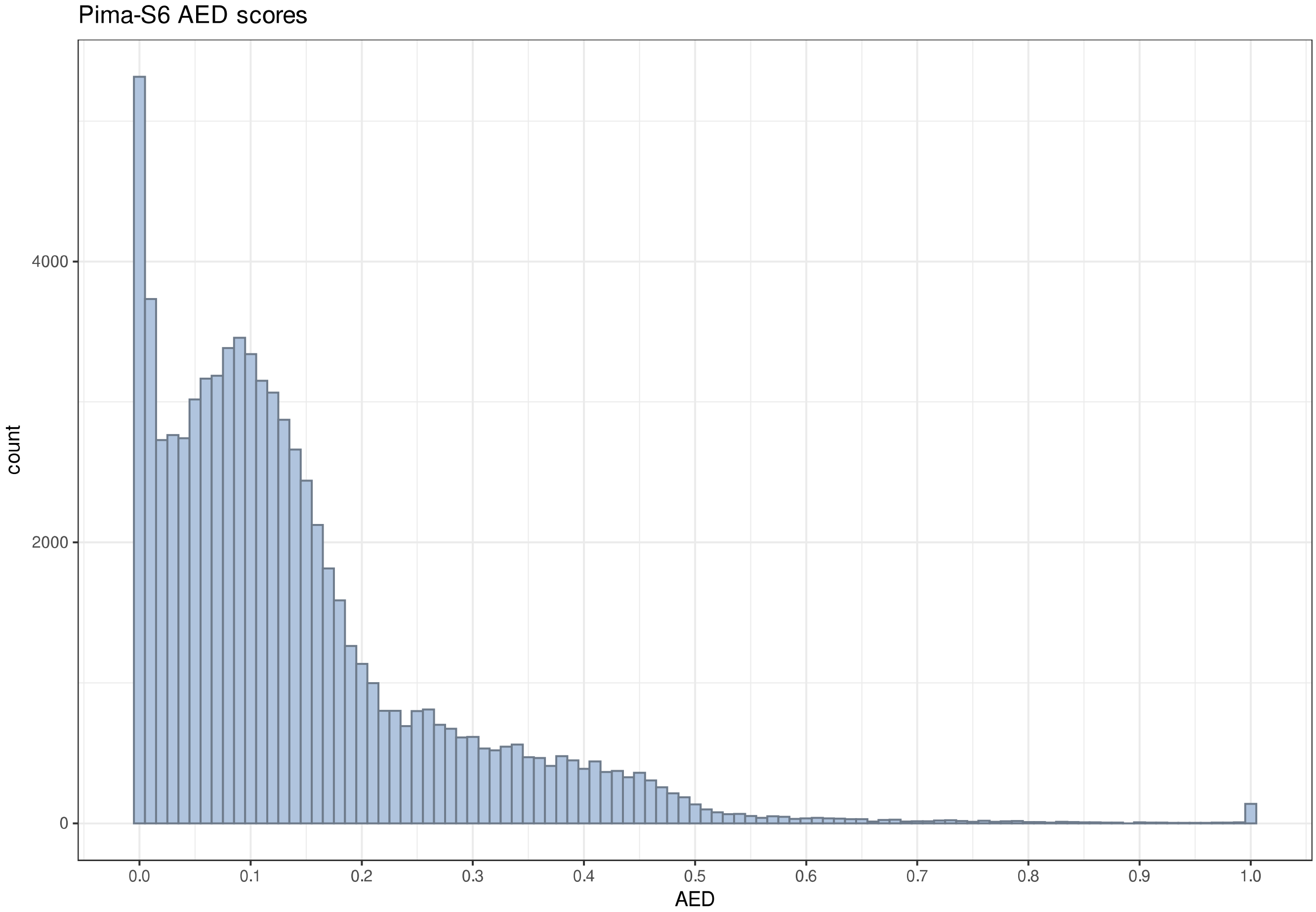

Supplementary Figure 1. Annotation Edit Distance scores of the Pima-S6 genome annotation. This histogram shows the distribution of Annotation Edit Distance (AED) scores, a measure of the goodness of fit of an annotation to the evidence supporting it, for the 75,419 genes in the final Pima-S6 annotation. From the MAKER/MAKER-P protocol (Campbell, M. S., Holt, C., Moore, B. and Yandell, M. 2014. Genome Annotation and Curation Using MAKER and MAKER-P. Curr. Protoc. Bioinform. 48:4.11.1-4.11.39.; doi: 10.1002/0471250953.bi0411s48): “AED is a number between 0 and 1, with an AED of zero denoting perfect concordance with the available evidence and a value of one indicating a complete absence of support for the annotated gene model. In other words, the AED score provides a measure of each annotated transcript’s congruency with its supporting evidence.”

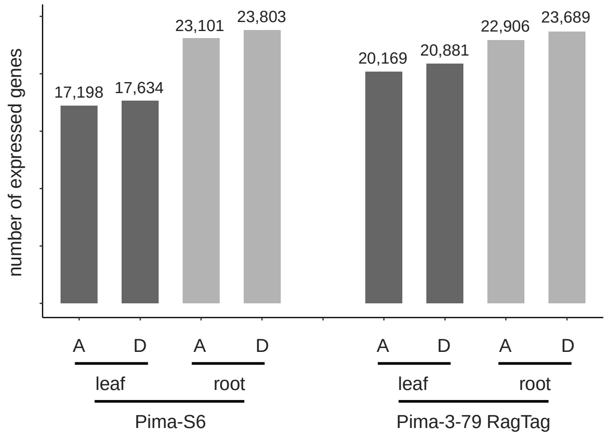

Supplementary Figure 2. Overview of leaf and root gene expression in Pima-S6 and Pima-3-79 RagTag. RNA from one leaves and one roots sample was isolated and sequenced, and gene expression was quantified. Bars represent the number of expressed genes (TPM > 1) in each species, organ and location on the A or D genome.

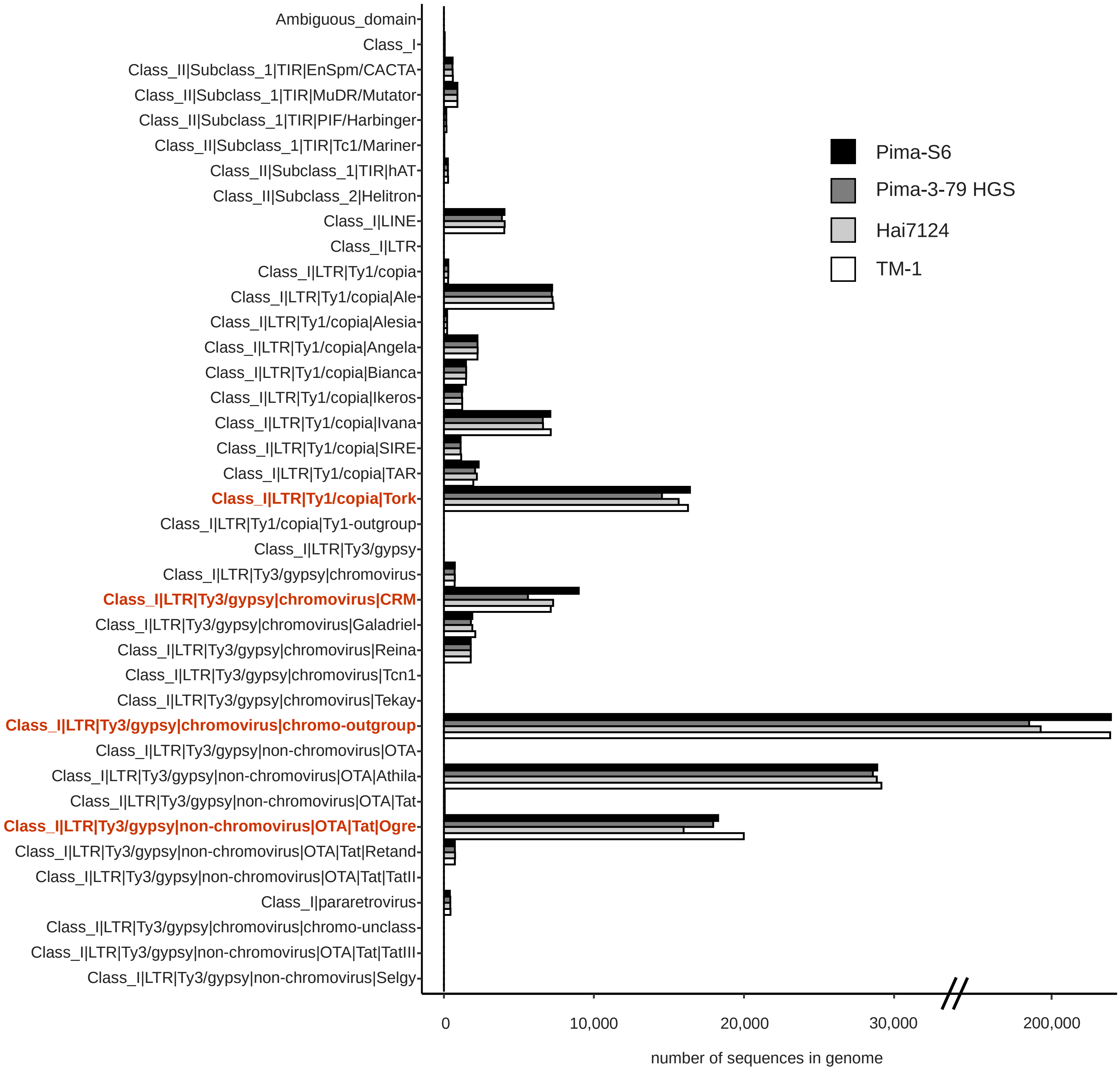

Supplementary Figure 3a. LTR retrotransposon families distribution if four Gossypium assemblies, A genome. The 13 A genome chromosomes from Pima-S6, Pima-3-79 HGS, Hai7124 and TM-1 were analyzed using the Domain based ANnotation of Transposable Elements (DANTE) tool and the REXdb Viridiplantae v3.0 database. The output file was filtered on the RepeatExplorer Galaxy server at https://repeatexplorer-elixir.cerit-sc.cz/ using default parameters, and the number of sequences per LTR retrotransposon family was plotted.

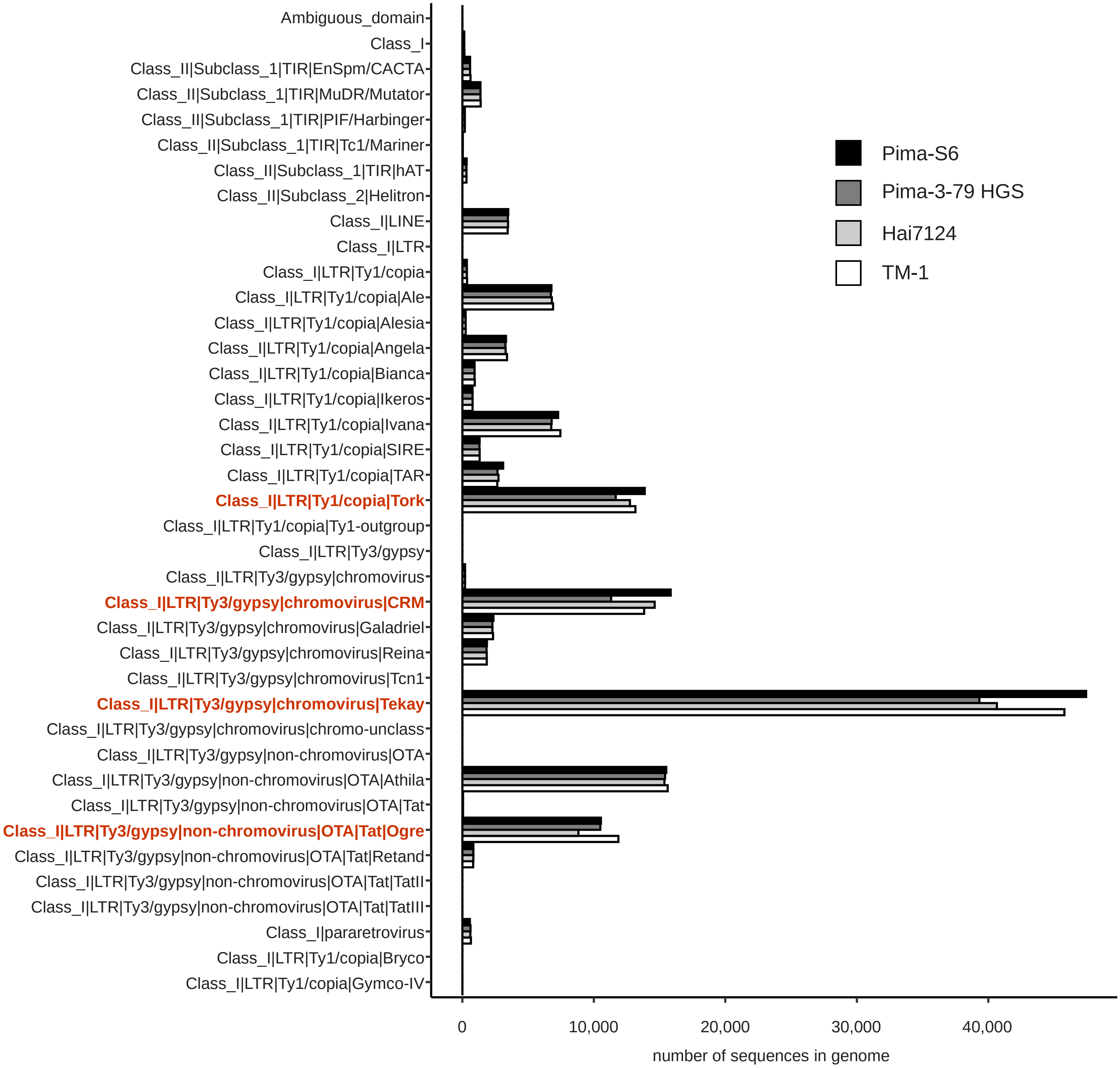

Supplementary Figure 3b. LTR retrotransposon families distribution if four Gossypium assemblies, D genome. The 13 D genome chromosomes from Pima-S6, Pima-3-79 HGS, Hai7124 and TM-1 were analyzed using the Domain based ANnotation of Transposable Elements (DANTE) tool and the REXdb Viridiplantae v3.0 database. The output file was filtered on the RepeatExplorer Galaxy server at https://repeatexplorer-elixir.cerit-sc.cz/ using default parameters, and the number of sequences per LTR retrotransposon family was plotted.

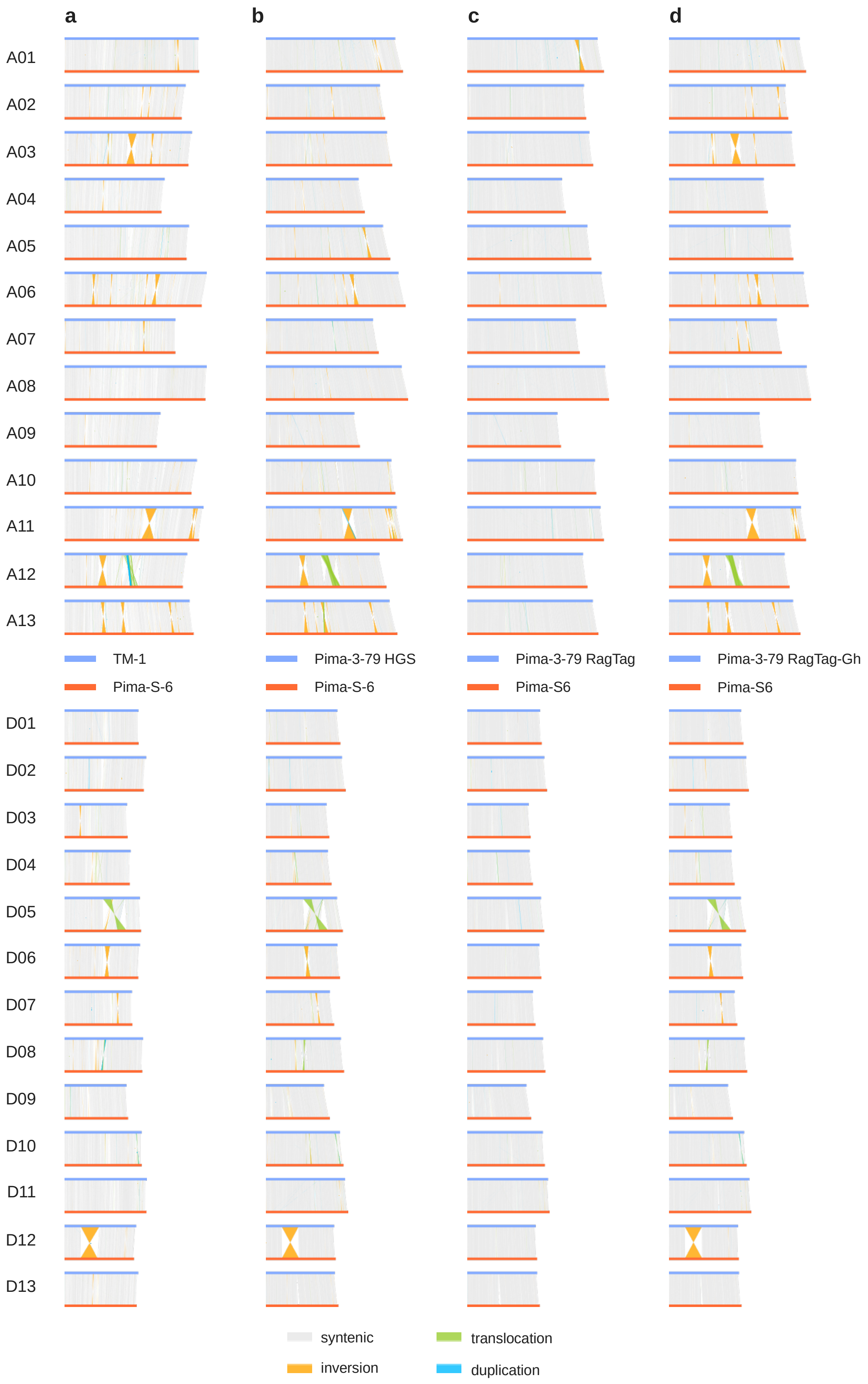

Supplementary Figure 4. Synteny plots between Pima-S6 and Pima-3-79 HGS, TM-1, Pima3-79 RagTag. and Pima3-79 RagTag-Gh In these synteny plots, the reference genome is represented by blue horizontal lines and the query genome by red horizontal lines. Vertical lines represent syntenic (grey), inverted (orange), translocated (green) and duplicated (blue) regions. Chromosome ids are indicated on the left side of the plots. The 26 chromosomes of our Pima-S6 assembly were aligned using minimap2 versus the 26 chromosomes of *G. hirsutum* TM-1 (**a**), Pima-3-79 HGS (**b**), Pima-3-79 HGS RagTag (our re-scaffolding of Pima-3-79 HGS using Pima-S6 as reference; **c**) or Pima-3-79 RagTag-Gh (our re-scaffolding of Pima-3-79 HGS using TM-1 as reference; **d**), and synteny analysis was carried out using syri. All major structural variations visible in the Pima-S6 vs TM-1 and Pima-S6 vs Pima-3-79 HGS plots are no longer present in the Pima-S6 vs Pima-3-79-RagTag plot, except for a ~4 Mbp inversion and ~0.5 Mbp duplication in chromosome A01, while the Pima-S6 vs Pima-3-79 RagTag-Gh plot recapitulates all the major chromosomal rearrangements from the Pima-S6 vs Pima-3-79 HGS plot.

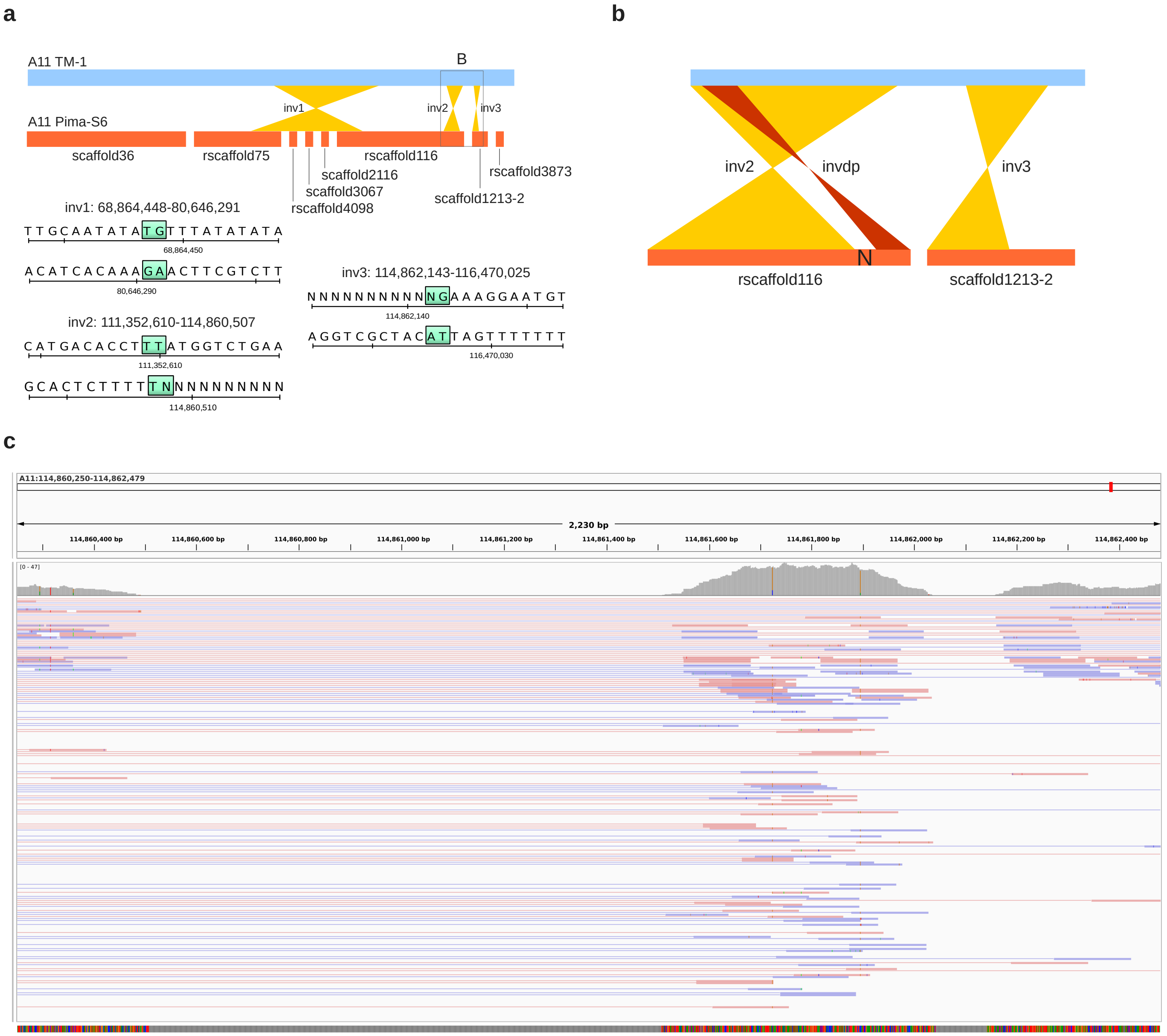

Supplementary Figure 5. Pima-S6 vs TM-1 chromosome A11 inversions. (**a**) Schematic representation of the chromosome A11 synteny. The TM-1 A11 chromosome is represented by a blue horizontal box and the scaffolds that form the Pima-S6 A11 chromosome are represented by red boxes. Scaffold ids are indicated below each box. The prefix “r” in a scaffold name indicates a scaffold placed in reverse orientation. Gaps between scaffolds represent the 100 bp N gaps introduced during chromosome reconstruction. The three major inversions (> 1 Mbp) are represented by orange hourglasses. For each inversion a zoom on the sequence at the inversion start and end is shown, with the exact inversion boundaries highlighted by green boxes. Inversion1's boundaries traverse unambiguously assembled regions. Inversion2 starts at an unambiguously assembled region and ends at the start of a 1,000 bp N stretch. Inversion3 starts at the beginning of scaffold1213-2 and ends at an unambiguously assembled region. (**b**) Detailed view of the inversion2 to inversion3 region. Inversion2 is followed by a 1,000 bp N stretch (represented by the letter N inside rscaffold116) and 535 bp of unambiguous sequence. These 535 bp are themselves an inversion-duplication (invdp) nested within inversion2 on the TM-1 side. (**c**) IGV screenshot of the rscaffold116 1,000 N region. Re-alignment of the mate-pair reads showed uniquely mapped reads (bowtie2 XS flag null and not duplicate filter) aligned to rscaffold116's 535 bp end with their mate thousands of bp upstream, confirming the linkage evidence across the 1,000 bp N stretch.

d

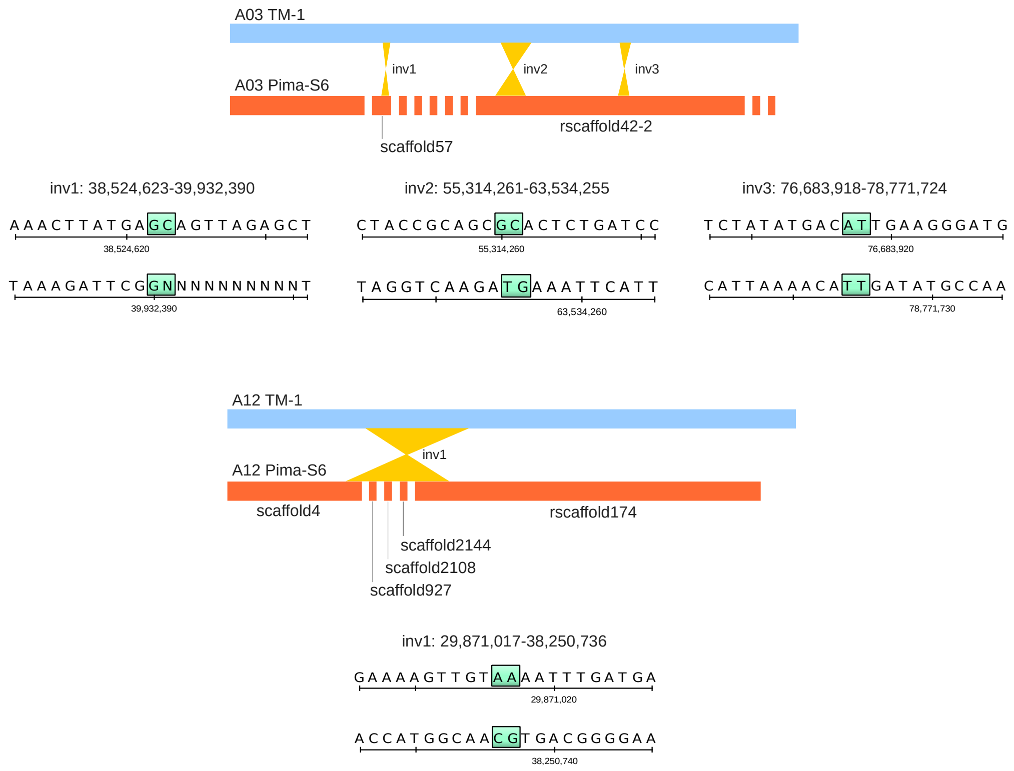

e

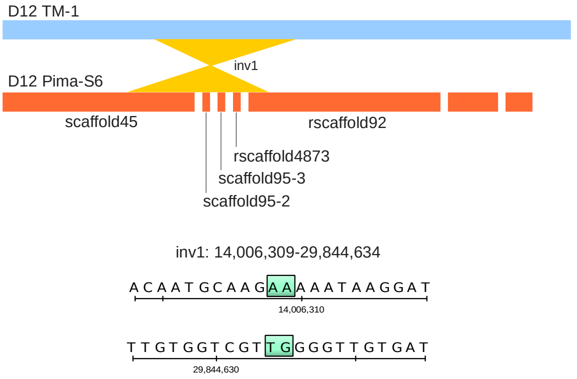

Supplementary Figure 5 (continued). TM-1 vs Pima-S6 synteny plots for chromosomes A03, A12 and D12. All inversion boundaires, except the end of inversion1 on chromosome A03, traverse unambiguously assembled regions. The 3' end of inversion1 on chromosome A03 is followed by a 10 bp N stretch and a 1,532 bp inversion-duplication of unambiguously assembled sequence that aligns to a chromosome A11 region on the TM-1 side.

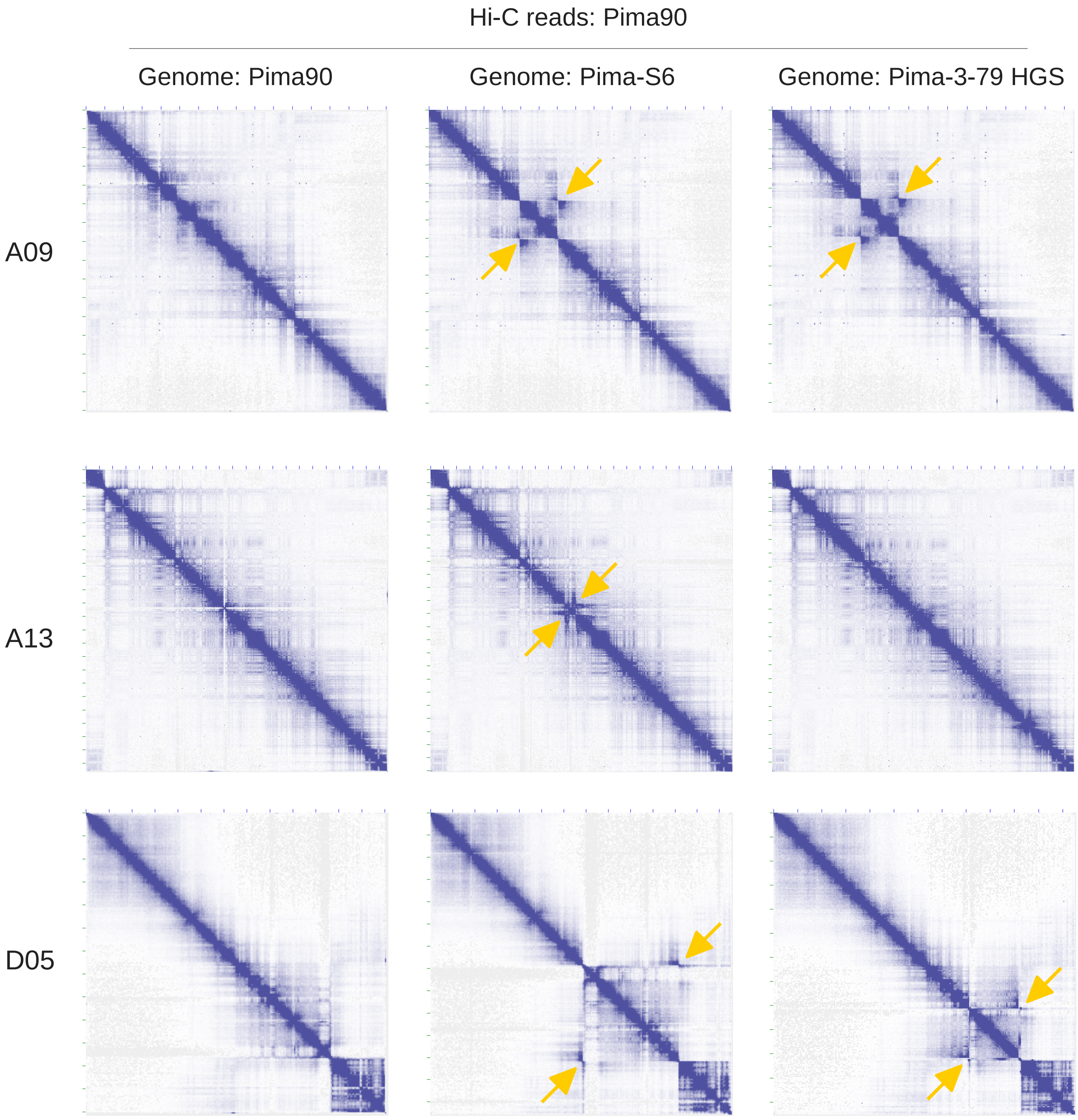

Supplementary Figure 6. Hi-C plots of Pima90 Hi-C reads aligned to the Pima90, Pima-S6 and Pima-3-79 HGS genomes. In these plots, the blue color indicates a chromatin contact. The coordinate 1 of each chromosome is located at the top left corner. Only chromosomes A09, A13 and D05 are shown. In the Pima-S6 plot, the inversions in these three chromosomes are clearly visible (orange arrows). In the Pima-3-79 HGS plot, the inversions in chromosomes A09 and D05 are also clearly visible, but an inversion in chromosome A13 is not apparent. The Pima90 plot is shown as reference.

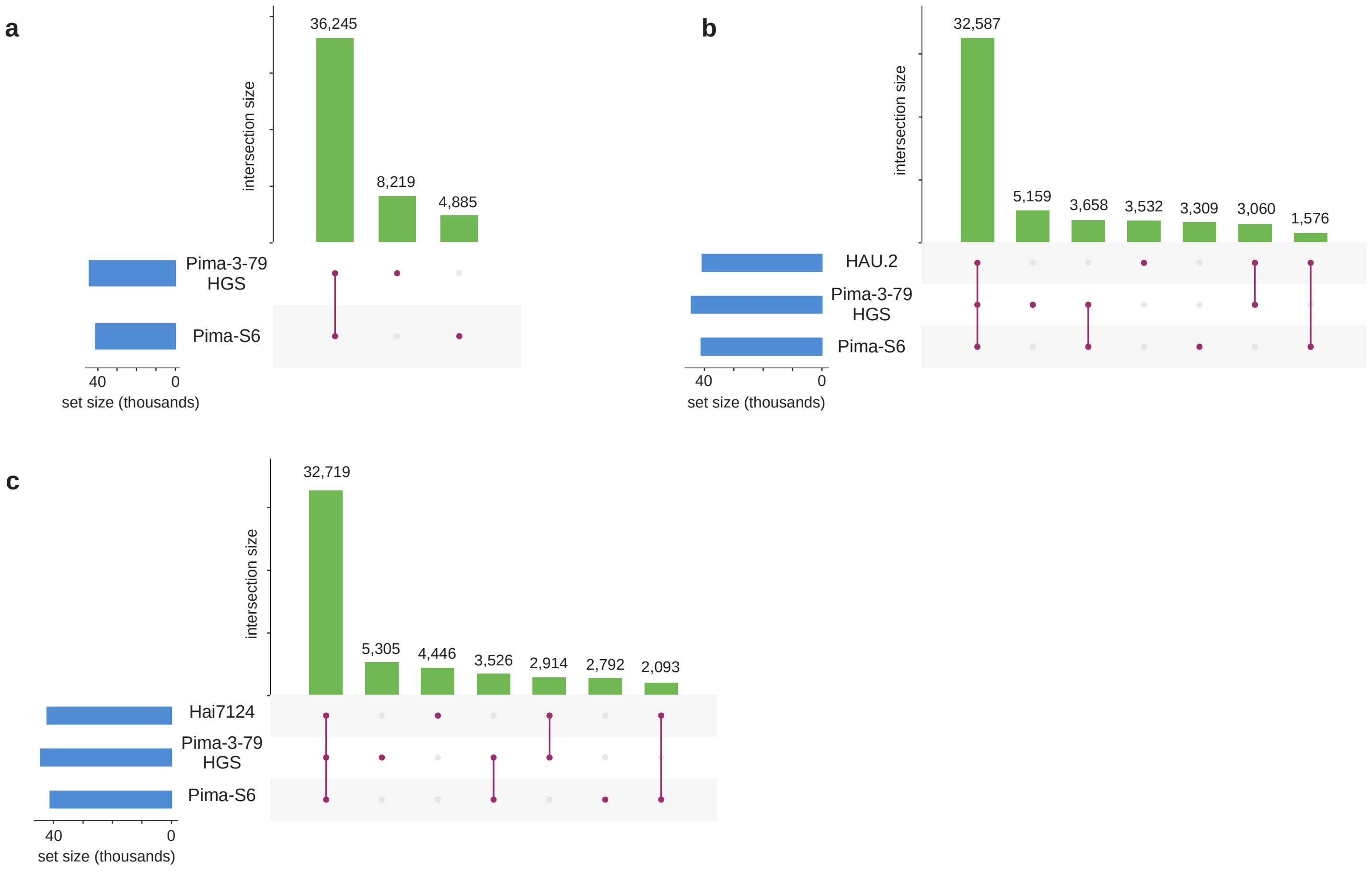

Supplementary Figure 7. Number of orthogroups shared between Pima-S6, Pima-3-79 HGS, Pima-3-79 HAU.2 and Hai7124. In these UpSet plots, set size indicates the number of orthogroups for each species, and intersection size indicates the number of orthogroups in each of the intersections shown below the corresponding bar. A total of 75,263 orthogroups were identified from the annotated proteins of *G. hirsutum* 'TM-1' (genome AD_1), *G. barbadense* 'Pima-S6' (AD_2; Pima-S6), *G. barbadense* 'Pima-3-79 HGS' (AD2; Pima-3-79 HGS), *G. barbadense* 'Pima-3-79' HAU.2 (AD2; HAU.2), *G. barbadense* Hai7124 (AD_2; Hai7124), *G. tomentosum* (AD3), *G. mustelinum* (AD4), *G. darwinii* (AD5), *G. herbaceum* (A1), *G. arboreum* (A2) and *G. raimondii* (D5). (**a**) Number of orthogroups shared between Pima-S6 and Pima-3-79 HGS. The number of shared orthogroups is lower than the number of shared orthogroups between Pima-S6 and Pima-3-79 RagTag in Figure 5a. (**b**) Number of orthogroups shared between three Pima assemblies, Pima-S6, Pima-3-79 HGS and Pima-3-79 HAU.2. Pima-3-79 HGS and Pima-3-79 HAU.2 have an important number of proteins without an ortholog in any of the other two assemblies. (**c**) Number of orthogroups shared between Pima-S6, Pima-3-79 HGS and Hai7124. Pima-3-79 HGS has the highest number of orthogroups without orthologs in Pima-S6 or Hai7124.

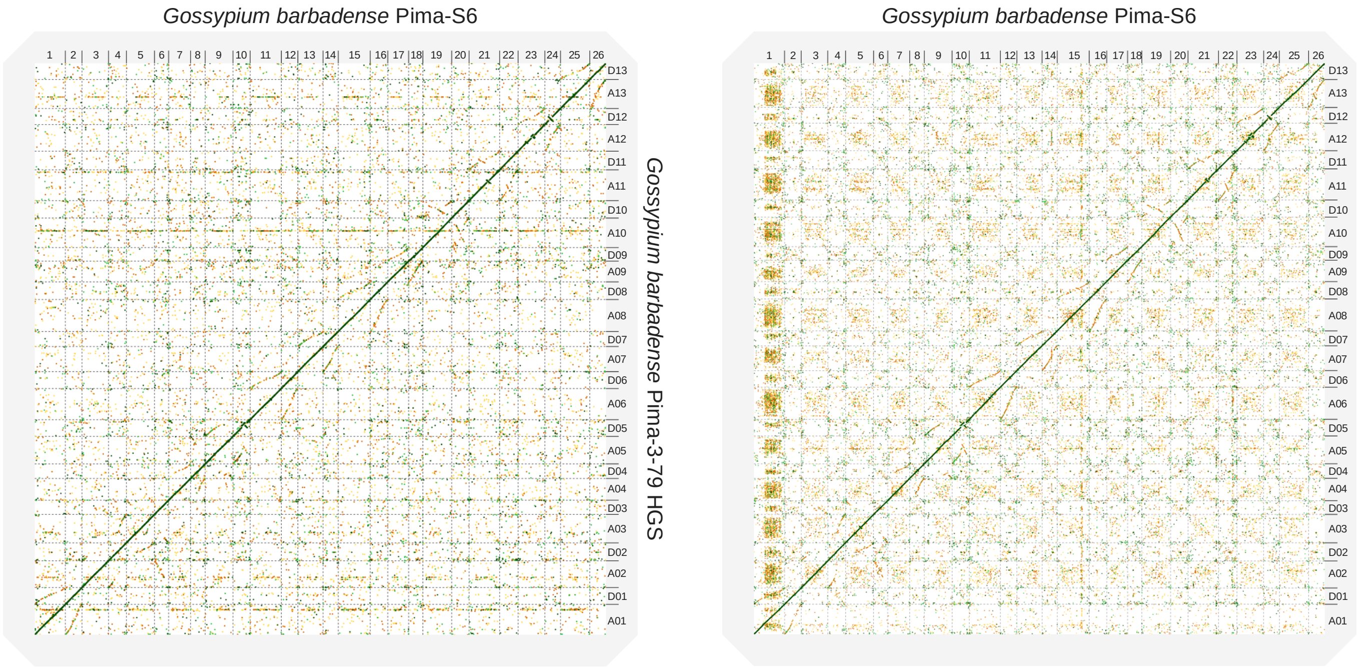

Supplementary Figure 8. Dot plots of Pima-S6 vs Pima-3-79 HGS and TM-1 genome assemblies alignments. The 26 chromosomes of the indicated genome assemblies were aligned using minimap2, and the PAF alignment file was plotted using D-Genies. Pima-S6 chromosome ids were renamed to the AD nomenclature using the best matching chromosome in Pima-3-79 HGS and TM-1: chromosome 1 was renamed A01, chromosome 2 D01, chromosome 3 A02, chromosome 4 D02, and so on.

Supplementary Table 1. Statistics for Pima-S6 and Pima-3-79 RNA-seq leaf and root RNA-seq samples.

Sample	Total reads	Aligned reads	Uniquely aligned reads	Aligned reads (%)	Uniquely aligned reads (%)
Pima-S6 leaf	4,105,123	2,858,840	2,407,125	69.6	58.6
Pima-S6 root	4,765,455	3,903,337	3,384,533	81.9	71.0
Pima-3-79 leaf	4,477,465	3,708,087	3,147,555	82.8	70.3
Pima-3-79 root	5,034,226	4,176,066	3,645,570	83.0	72.4

Supplementary Table 2. RepeatExplorer analysis of the TM-1, Pima-3-79 HGS, Hai7124 and Pima-S6 A and D genomes. The two tables contain the filtered counts output of RepeatExplorer for the A (this table) and D genomes (table below). Families mentioned in the manuscript are highlighted bold.

A genome

CLASSIFICATION	TM-1	Pima-3-79 HGS	Hai7124	Pima-S6
Ambiguous_domain	0	0	0	0
Class_I	64	60	59	65
Class_II|Subclass_1|TIR|EnSpm/CACTA	596	584	592	591
Class_II|Subclass_1|TIR|MuDR/Mutator	911	902	911	906
Class_II|Subclass_1|TIR|PIF/Harbinger	175	163	164	164
Class_II|Subclass_1|TIR|Tc1/Mariner	29	29	30	30
Class_II|Subclass_1|TIR|hAT	277	275	271	276
Class_II|Subclass_2|Helitron	3	3	2	3
Class_I|LINE	4,026	3,867	4,058	4,048
Class_I|LTR	0	0	0	0
Class_I|LTR|Ty1/copia	288	296	297	297
Class_I|LTR|Ty1/copia|Ale	7,316	7,188	7,248	7,219
Class_I|LTR|Ty1/copia|Alesia	219	214	219	217
Class_I|LTR|Ty1/copia|Angela	2,248	2,247	2,261	2,245
Class_I|LTR|Ty1/copia|Bianca	1,479	1,482	1,488	1,480
Class_I|LTR|Ty1/copia|Ikeros	1,220	1,204	1,229	1,233
Class_I|LTR|Ty1/copia|Ivana	7,131	6,589	6,613	7,092
Class_I|LTR|Ty1/copia|SIRE	1,156	1,118	1,119	1,117
Class_I|LTR|Ty1/copia|TAR	1,969	2,089	2,199	2,318
**Class_I|LTR|Ty1/copia|Tork**	**16,270**	**14,535**	**15,647**	**16,393**
Class_I|LTR|Ty1/copia|Ty1-outgroup	0	0	0	0
Class_I|LTR|Ty3/gypsy	0	0	0	0
Class_I|LTR|Ty3/gypsy|chromovirus	716	717	728	741
**Class_I|LTR|Ty3/gypsy|chromovirus|CRM**	**7,123**	**5,599**	**7,289**	**8,995**
Class_I|LTR|Ty3/gypsy|chromovirus|Galadriel	2,097	1,812	1,894	1,904
Class_I|LTR|Ty3/gypsy|chromovirus|Reina	1,794	1,793	1,789	1,793
Class_I|LTR|Ty3/gypsy|chromovirus|Tcn1	0	0	0	0
**Class_I|LTR|Ty3/gypsy|chromovirus|Tekay**	**219,867**	**193,125**	**196,891**	**220,150**
Class_I|LTR|Ty3/gypsy|chromovirus|chromo-outgroup	0	0	0	0
Class_I|LTR|Ty3/gypsy|non-chromovirus|OTA	0	0	0	0
Class_I|LTR|Ty3/gypsy|non-chromovirus|OTA|Athila	29,151	28,589	28,848	28,875
Class_I|LTR|Ty3/gypsy|non-chromovirus|OTA|Tat	52	57	56	58
Class_I|LTR|Ty3/gypsy|non-chromovirus|OTA|Tat|Ogre	19,984	17,944	15,973	18,279
Class_I|LTR|Ty3/gypsy|non-chromovirus|OTA|Tat|Retand	737	733	748	741
Class_I|LTR|Ty3/gypsy|non-chromovirus|OTA|Tat|TatII	0	0	0	0
Class_I|pararetrovirus	448	435	424	402
Class_I|LTR|Ty3/gypsy|chromovirus|chromo-unclass	0	0	0	0
Class_I|LTR|Ty3/gypsy|non-chromovirus|OTA|Tat|TatIII	0	0	0	0
Class_I|LTR|Ty3/gypsy|non-chromovirus|Selgy	0	0	0	0

Supplementary Table 2 (contiinued).

D genome

CLASSIFICATION	TM-1	Pima-3-79 HGS	Hai7124	Pima-S6
Ambiguous_domain	0	0	0	0
Class_I	159	134	138	140
Class_II|Subclass_1|TIR|EnSpm/CACTA	609	591	594	591
Class_II|Subclass_1|TIR|MuDR/Mutator	1,388	1,377	1,374	1,376
Class_II|Subclass_1|TIR|PIF/Harbinger	186	184	184	190
Class_II|Subclass_1|TIR|Tc1/Mariner	30	29	30	30
Class_II|Subclass_1|TIR|hAT	312	312	311	316
Class_II|Subclass_2|Helitron	4	4	4	4
Class_I|LINE	3,436	3,452	3,478	3,486
Class_I|LTR	0	1	1	1
Class_I|LTR|Ty1/copia	351	338	338	342
Class_I|LTR|Ty1/copia|Ale	6,890	6,733	6,808	6,774
Class_I|LTR|Ty1/copia|Alesia	251	252	242	250
Class_I|LTR|Ty1/copia|Angela	3,396	3,274	3,291	3,324
Class_I|LTR|Ty1/copia|Bianca	946	929	925	928
Class_I|LTR|Ty1/copia|Ikeros	775	769	775	766
Class_I|LTR|Ty1/copia|Ivana	7,449	6,791	6,758	7,285
Class_I|LTR|Ty1/copia|SIRE	1,312	1,297	1,309	1,307
Class_I|LTR|Ty1/copia|TAR	2,649	2,672	2,747	3,099
**Class_I|LTR|Ty1/copia|Tork**	**13,156**	**11,671**	**12,744**	**13,864**
Class_I|LTR|Ty1/copia|Ty1-outgroup	0	0	0	0
Class_I|LTR|Ty3/gypsy	2	1	1	1
Class_I|LTR|Ty3/gypsy|chromovirus	209	207	209	208
**Class_I|LTR|Ty3/gypsy|chromovirus|CRM**	**13,824**	**11,307**	**14,621**	**15,836**
Class_I|LTR|Ty3/gypsy|chromovirus|Galadriel	2,334	2,286	2,277	2,363
Class_I|LTR|Ty3/gypsy|chromovirus|Reina	1,847	1,837	1,856	1,860
Class_I|LTR|Ty3/gypsy|chromovirus|Tcn1	0	0	0	0
**Class_I|LTR|Ty3/gypsy|chromovirus|Tekay**	**45,785**	**39,324**	**40,653**	**47,442**
Class_I|LTR|Ty3/gypsy|chromovirus|chromo-unclass	0	0	0	0
Class_I|LTR|Ty3/gypsy|non-chromovirus|OTA	0	1	1	1
Class_I|LTR|Ty3/gypsy|non-chromovirus|OTA|Athila	15,613	15,408	15,370	15,509
Class_I|LTR|Ty3/gypsy|non-chromovirus|OTA|Tat	46	51	50	51
Class_I|LTR|Ty3/gypsy|non-chromovirus|OTA|Tat|Ogre	11,865	10,491	8,818	10,531
Class_I|LTR|Ty3/gypsy|non-chromovirus|OTA|Tat|Retand	826	834	833	838
Class_I|LTR|Ty3/gypsy|non-chromovirus|OTA|Tat|TatII	0	0	0	0
Class_I|LTR|Ty3/gypsy|non-chromovirus|OTA|Tat|TatIII	0	0	0	0
Class_I|pararetrovirus	651	619	596	576
Class_I|LTR|Ty1/copia|Bryco	0	0	0	0
Class_I|LTR|Ty1/copia|Gymco-IV	0	0	0	0

Supplementary Table 3. *G. barbadense* assemblies chromosome lenghts. Chromosome lengths, in bp, of the Hai7124, Pima90, Pima-3-79 HAU.1, Pima-3-79 HAU.2, Pima-3-79 HGS, and Pima-3-79 HGS-RagTag (our re-scaffolding of the Pima-3-79 HGS assembly) and Pima-S6 assemblies.

Chromosome	Hai7124	Pima90	Pima-3-79 HAU.1	Pima-3-79 HAU.2	Pima-3-79 HGS	Pima-3-79 HGS-RagTag	Pima-S6
A01	115,639,457	116,205,217	83,454,191	115,637,255	113,238,469	114,571,553	120,119,894
A02	102,048,560	103,411,127	89,731,153	100,057,689	99,769,429	102,453,845	104,444,548
A03	107,689,529	108,114,255	102,275,589	105,315,579	105,981,974	107,161,519	110,526,773
A04	82,003,869	83,632,351	66,299,034	81,554,553	80,954,414	82,930,467	86,331,418
A05	107,778,512	105,775,400	93,810,923	102,776,486	102,458,744	105,538,069	108,864,071
A06	118,151,758	119,984,783	100,034,992	115,140,250	116,119,172	118,127,218	122,525,983
A07	95,011,077	98,954,622	87,365,090	92,880,876	93,754,744	95,177,537	98,767,759
A08	120,787,428	121,356,893	108,674,945	119,882,356	119,114,718	121,341,730	125,840,848
A09	79,226,513	80,060,025	79,449,896	77,927,517	77,140,527	78,896,073	82,042,076
A10	111,721,976	111,536,923	107,075,239	110,302,803	109,871,119	112,125,172	113,317,428
A11	116,189,812	116,982,619	96,770,036	113,101,708	114,694,469	117,157,560	120,065,767
A12	101,615,779	102,448,369	93,480,475	102,106,374	99,282,030	101,695,573	105,494,938
A13	112,976,367	112,994,790	86,578,892	109,233,746	108,235,369	110,287,702	115,221,231
**A genome**	**1,370,840,637**	**1,381,457,374**	**1,195,000,455**	**1,345,917,192**	**1,340,615,178**	**1,367,464,018**	**1,413,562,734**
D01	63,619,354	64,421,210	62,968,913	62,811,768	62,611,448	63,857,906	65,361,719
D02	68,884,445	69,736,045	71,317,061	67,659,327	66,877,699	67,695,607	70,102,368
D03	53,779,058	54,629,598	48,575,973	50,986,338	53,043,351	53,687,303	55,503,051
D04	57,348,712	57,514,160	51,938,909	52,479,919	54,342,187	54,661,041	57,477,808
D05	66,507,725	65,267,064	60,343,712	63,323,194	62,429,648	64,737,037	67,540,788
D06	62,791,478	64,267,889	66,700,274	62,964,862	62,820,931	63,351,879	65,076,424
D07	57,193,027	58,605,821	61,301,369	56,457,815	56,075,531	57,709,291	59,768,398
D08	67,735,652	68,772,276	69,128,419	65,987,480	65,831,471	66,566,626	68,651,644
D09	53,804,133	54,849,252	53,850,888	51,526,817	50,685,742	51,740,324	55,929,093
D10	66,844,369	67,066,849	64,637,854	65,706,892	65,066,651	66,412,153	68,149,462
D11	72,283,341	73,674,071	65,624,333	68,311,261	69,577,400	71,006,005	72,385,828
D12	60,787,228	61,669,802	63,542,810	58,873,029	59,787,526	59,929,623	61,230,550
D13	61,297,484	62,736,228	62,378,783	60,343,961	60,421,729	61,273,340	63,610,372
**D genome**	**812,876,006**	**823,210,265**	**802,309,298**	**787,432,663**	**789,571,314**	**802,628,135**	**830,787,505**
**26 chromosomes**	**2,183,716,643**	**2,204,667,639**	**1,997,309,753**	**2,133,349,855**	**2,130,186,492**	**2,170,092,153**	**2,244,350,239**

Supplementary Table 4. Pima-S6 genomic sequencing libraries statistics.

Library type	Insert size	Sequencing	Number of libraries	Number of reads	Approximate depth
PCR-free paired-end	440-470 bp	2 x 250	1	370655026	160x
Mate-pair (Nextera™ MP Gel Plus)	2-4 kbp	2 x 150	1	222776057	58x
10X genomics™ Chromium ™	NA	2 x 150	1	315583626	82x
